# Dynamics of the time-fractional reaction–diffusion coupled equations in biological and chemical processes

**DOI:** 10.1038/s41598-024-58073-z

**Published:** 2024-03-30

**Authors:** Abdul Ghafoor, Muhammad Fiaz, Manzoor Hussain, Asad Ullah, Emad A. A. Ismail, Fuad A. Awwad

**Affiliations:** 1 Institute of Numerical Sciences, Kohat University of Science and Technology, Kohat, 26000 KP Pakistan; 2 Department of Mathematics, Faculty of Sciences and Technology, Women University of Azad Jammu and Kashmir, Bagh, Azad Kashmir Pakistan; 3 School of Finance and Economics, Jiangsu University, 301, Xuefu Road, Jingkou District, Zhenjiang, 212013 Jiangsu China; 4 Department of Mathematical Sciences, University of Lakki Marwat, Lakki Marwat, 28420 Khyber Pakhtunkhwa Pakistan; 5 Department of Quantitative Analysis, College of Business Administration, King Saud University, P.O. Box 71115, Riyadh, 11587 Saudi Arabia

**Keywords:** Fractional calculus, Implicit scheme, Caputo fractional derivative, Brusselator model, Schnakenberg model, Gray–Scott model, Stability analysis, Energy science and technology, Mathematics and computing

## Abstract

This paper aims to demonstrate a numerical strategy via finite difference formulations for time fractional reaction–diffusion models which are ubiquitous in chemical and biological phenomena. The time-fractional derivative is considered in the Caputo sense for both linear and nonlinear problems. First, the Caputo derivative is replaced with a quadrature formula, then an implicit method is used for the remaining part. In the linear case, the proposed strategy reduces the time fractional models into linear simultaneous equations. In nonlinear cases, Quasilinearization is utilized to tackle the nonlinear parts. With this strategy, solutions of the fractional system transform into linear algebraic systems which are easy to solve. Next, the Von Neumann method is implemented to examine the stability of the scheme which discloses that the scheme is unconditionally stable. Further, the applicability of the presented scheme is tested with different linear and nonlinear models which include the one dimensional Schnakenberg and Gray–Scott models, and one and two dimensional Brusselator models. To analyze the accuracy of the present technique two norms namely, $\mathbb {L}_{\infty }$ and $\mathbb {L}_{2}$, and relative error are addressed. Moreover, the obtained outcomes are shown tabulated and graphically which identifies that the scheme properly works for the time fractional reaction–diffusion systems.

## Introduction

Reaction–diffusion models (RDMs) play a vital role in describing various spatial patterns like mazes, stripes, and spots through chemical operations in cells. RDMs theory has been started from the pioneer work of Turing^1^ which explored the importance of pattern formation via RDMs and biological processes. Particularly, this theory describes that uniform stability of the system remains in the absence of diffusion parameters while different spatial pattern formations can be realized in the presence of reaction and diffusion. Many authors identified the usage of RDMs models in various scientific and engineering disciplines. For example pattern formation in hydra^2^, shell pigmentation^3^, animal coat markings^4^ and many other for which the readers may refer to see^5^. The aforementioned applications show that RDMs are ubiquitous in different areas of science.

RDMs are highly non-linear and its closed form solution is a challenging task. Therefore, numerical techniques are the alternative remedies to capture the dynamics of non-linear models. Several computational strategies have been advised in the literature to determine the numerical solutions of non-linear RDMs related to pattern formation. For example, Ersoy^6^ established a computational algorithm for the study of RDMs using an exponential cubic B-spline. Onarcan et al.^7^ proposed a numerical based on trigonometric cubic B-spline to solve RDMs. Similarly, finite difference-based techniques^8,9^ and finite element method^10^ have been used to solve the RDMs. Mittal and his co-author developed solved RDMs by modified cubic B-spline coupled with differential quadrature. Korkmaz et al.^11^ investigated the motion of different patterns modeled by a special case of RDMs.

Different RDMs are presented in available studies. For example, Brusselator model which is also known as tri-molecular chemical reaction system which demonstrates the existence of Turing instability in the autocatalytic reactions^12^. Another famous system is the Gray-Scott model which delineates spot-like patterns that remain self-reproducing structures in the whole domain^11^. Besides, RDMs consist of the well-known Schnakenberg model also known as activator-depleted model. Physically, the Schnakenberg model elucidates a chemical reaction between the chemical substances namely: activator and an inhibitor. The fast disappearance of inhibitor than activator leads to Turing instability.

The study of the aforementioned models was limited to integer calculus which means that all RDMs involve integer order derivatives. The fractional version of these models is still an open area of research. In this case, the order of the involved derivatives is arbitrary which lies in well known branch of mathematics known as factional calculus(FC)^13^. The theory of FC was commenced when Leibnitz asked L’Hôpital about the half derivative and then the idea was extended in different directions by renowned mathematicians like Riemann, Abel, Laplace, and Euler. Recently many applications of fractional partial differential equations (FPDEs) have been observed in different fields of science and engineering such as bioengineering^14^, solid mechanics, nonlinear oscillations of an earthquake, fluid-dynamic traffic model^15^, economics, and anomalous transport^16^. Fractional models dominate over classical models in the sense of memory effect which means that the next stat of the dynamical system will equally defend on the preceding and present stats. FPDEs are subdivided into three categories, those having space fractional derivatives are known as space fractional models. Similarly, those having time fractional derivatives and combined space and time fractional derivatives are called time-fractional and space-time fractional models. Further, the FPDEs are divided on the basis of order which is either constant or a function of space and time variables which are called consultant and variable order FPDEs, respectively. In the last two decades, the time fractional partial differential equations (TFPDEs) attracted a lot of researchers due to the time evolution in fractions.

Numerous approaches have been discussed in existing work to solve FPDEs and TFPDEs. Such as, Chen et al.^17^ presented fractional percolation problem via implicit finite difference method. Huang and Zhao^18^ implemented two distinct alternating direction implicit numerical stratagems for the numerical estimation of linear non-linear super diffusion equations. In^19^ finite element methods (FEMs) have been suggested to solve TFPDEs like fractional advection–dispersion systems, and Fokker–Planck model^20^ having fractional time and space derivatives. Like finite difference techniques and FEMs, Meshfree numerical procedures have been implemented for FPDEs and TFPDEs. For example, Uddin^21^ explored radial basis functions (RBFs) meshfree strategy for the numerical simulations of TFPDEs. Hussain et al.^22^ solved the time-fractional coupled KdV equations via meshless spectral numerical technique. Aghdam et al.^23^ utilized shifted Legendre polynomials and proposed a computational strategy for the numerical estimation of fractional advection–diffusion equations. Similarly, in^24^ the authors developed an efficient method to simulate the fractional Black-Scholes model for European options via Gegenbauer polynomials and Caputo derivatives. Moreover, a higher order numerical scheme based on quadratic interpolation for time and Chebyshev collocation for space was addressed for the solutions of space-time fractional advection–diffusion equation^25^. Mesgarani et al.^26^ proposed a numerical scheme for the space fractional advection–diffusion equation b coupling second-order accurate difference approach and second kind shifted Chebyshev polynomials.

Besides, we address some more recent work related to pattern formation. Such as Owolabi et al.^27^ proposed Fourier spectral method for the study of unveil complex Turing patterns arising in autocatalytic reactions via the Caputo fractional derivative. Alqhtani et al.^28^ investigated spatiotemporal and chaotic patterns with fractional order prey-predator models. Alqhtani et al.^29^ studied the Caputo fractional derivatives in predator-prey models to explore, the formation of spatiotemporal patterns through local and global stability analysis. The authors in^30^ presented different pattern formation scenarios of various superdiffusive system involving Caputo operator. Owolabi et al.^31^ developed a mathematical model using fractional-order super-diffusive processes for some emergent pattern formation in predator-prey system. Owolabi and Baleanu^32^ described several emergent patterns for diffusive turing-like models with fractional operator. Similarly in^33^ the authors studied the spatial patterns of modified prey-predator via diffusion-driven instability with associated chaotic behaviors. Moreover, Owolabi and Patidar^34^ discussed the higher order solutions strategies for the stiff time-dependent PDEs and its spatiotemporal dynamics of reaction–diffusion systems. Owolabi^35^ explored the pattern formation of different fractional reaction–diffusion systems. Pindza and Owolabi^36^ implemented a numerical scheme for the space fractional reaction–diffusion equations using Fourier spectral strategy for space and exponential integrator for time.

The current paper addresses the numerical solutions of time-fractional reactional models TFRDMs. There is sufficient space to study the dynamics of the TFRDMs. Our target is to consider these models in fractional form and explain the solutions numerically. The next goal is to elaborate on the stability analysis of the system. For verification of the proposed scheme, some test problems will be addressed. In those problems having an exact solution will be treated as artificial solutions for fractional cases. Moreover, three types of boundary conditions will be encountered.

The leftover paper is prescribed the following way. In “Basic definitions” some preliminaries related to FC, are reported. The proposed methodology and the boundary conditions are presented in “Description of the method for one-dimensional TFRDMs”. The stability of the scheme is given in “Stability analysis”. Algorithm for the proposed method is presented in “Algorithm”. Finally, the test paradigms and the conclusions are drawn in “Numerical experiments” and “Description of the method for two-dimensional TFRDMs”, respectively.

## Basic definitions

In this section of the manuscript, some basic definitions related to FC are discussed.

### Definition

The fractional derivative of $\chi$ in Caputo sense^37^ is defined as follow:

1 $$\begin{aligned} ~^{c} \mathscr {D}_{t}^{\alpha } \chi (x,t)= \frac{\partial ^{\alpha } \chi (x,t)}{\partial t^{\alpha }}= \left\{ \begin{array}{ll} \frac{1}{\Gamma (1-\alpha )} \int _0^t \frac{\partial \chi (x,s)}{\partial s}(t-s)^{-\alpha }ds, &{} 0< \alpha <1 \\ \\ \frac{\partial \chi }{\partial t}, &{} \alpha =1. \end{array} \right. \end{aligned}$$

### Quadrature rule

The Caputo fractional derivative of $\chi$ can be approximated by the quadrature formula given by^21^:

2 $$\begin{aligned} \frac{\partial ^{\alpha } \chi (x,t^{n+1})}{\partial t^{\alpha }} = \left\{ \begin{array}{ll} \mathscr {A}_{\alpha } \sum _{k=0}^{n} \beta ^{\alpha }_k (\chi ^{n-k+1} - \chi ^{n-k})+\mathscr {O}(\tau ^{2-\alpha }), &{} \text{ if } 0<\alpha < 1,\\ \frac{\chi ^{n+1}-\chi ^{n}}{\tau }+\mathscr {O}(\tau ) &{} \text{ if } \alpha =1, \end{array} \right. \end{aligned}$$

Where $\chi (x,t^{n})=\chi ^{n}$, $\mathscr {A}_{\alpha }=\frac{{\tau }^{-\alpha }}{\Gamma (2-\alpha )}$; $\tau$ is time step and $\beta ^{\alpha }_{k}=(k+1)^{1-\alpha }-(k)^{1-\alpha }$.

### Definition

The one and two parameters Mittag-Leffler function is defined as follows^37^:

3 $$\begin{aligned} \mathbb {E}_\eta (z)&= \sum _{k=0}^{\infty } {\frac{z^{k}}{\Gamma (\eta k+1)}} \qquad \eta >0, \end{aligned}$$

4 $$\begin{aligned} \mathbb {E}_{\eta ,\zeta } (z)&= \sum _{k=0}^{\infty } {\frac{z^{k}}{\Gamma (\eta k+\zeta )}} \qquad {\eta ,\zeta }>0. \end{aligned}$$

The Caputo derivative of exponential and trigonometric functions can be expressed in the form of the Mittag-Leffler function which is described below:

- $$~^{\mathscr {C}}_{0}\mathscr {D}_{x}^{\alpha }(e^{\lambda x})= \lambda ^{n} x^{n-\alpha } \mathbb {E}_{1,n-\alpha +1}(\lambda x)$$, where $$n=\lceil \alpha \rceil $$
- $$~^{\mathscr {C}}_{0}\mathscr {D}_{x}^{\alpha }sin({\lambda x})=\frac{1}{2i}((i \lambda )^{n} x^{n-\alpha } \mathbb {E}_{1,n-\alpha +1}(i \lambda x) - (i \lambda )^{n} x^{n-\alpha } \mathbb {E}_{1,n-\alpha +1}(i \lambda x) ) $$, where $$n=\lceil \alpha \rceil $$
- $$~^{\mathscr {C}}_{0}\mathscr {D}_{x}^{\alpha }cos({\lambda x})=\frac{1}{2}((i \lambda )^{n} x^{n-\alpha } \mathbb {E}_{1,n-\alpha +1}(i \lambda x) + (i \lambda )^{n} x^{n-\alpha } \mathbb {E}_{1,n-\alpha +1}(i \lambda x) )$$ , where $$n=\lceil \alpha \rceil $$.

## Description of the method for one-dimensional TFRDMs

Consider the following TFRDM^38–41^:

5 $$\begin{aligned}&\frac{\partial ^{\alpha } \mathscr {U}(x,t)}{\partial t^{\alpha }}=a_{1} \mathscr {U}_{xx} + b_{1} \mathscr {U} + c_{1} \mathscr {V} + d_{1} \mathscr {U}^{2} \mathscr {V} + e_1 \mathscr {U}\mathscr {V} + m_{1} \mathscr{U}\mathscr{V}^2 + \kappa _1 +\mathscr {F}_{1} (x,t),~x \in \Omega ,~ t> 0 \nonumber \\&\frac{\partial ^{\alpha } \mathscr {V}(x,t)}{\partial t^{\alpha }}=a_{2} \mathscr {V}_{xx} + b_{2} \mathscr {U} + c_{2} \mathscr {V} + d_{2} \mathscr {U}^{2} \mathscr {V} + e_{2} \mathscr{U}\mathscr{V} + m_{2} \mathscr{U}\mathscr{V}^2 + \kappa _2+\mathscr {F}_{2} (x,t),~x \in \Omega ,~ t > 0, \end{aligned}$$

In Eq. (5) $\mathscr {U}(x,t)$ and $\mathscr {V}(x,t)$ are two dependent variables describes the dynamics where $0<\alpha \le 1$, $a_{j} , b_{j} , c_{j} , d_{j}, e_{j} , m_{j}$ and $\kappa _j$, are real constants for each $j=1,2$ which described some physical interpretation discussed in problem 5.1. $\mathscr {F}_{1}$ and $\mathscr {F}_{2}$ are the source terms and $\Omega =[x_\textbf{0}, x_{N}]$ is the spacial domain is divided into M subintervals where width of each subinterval is $d {x} = \frac{{x_N}-{x_0}}{M}$. The associated initial conditions (ICs) are:

6 $$\begin{aligned} \mathscr {U}(x,0)=\mathscr {U}_{0}(x), \mathscr {V}(x,0)=\mathscr {V}_{0}(x)\quad x\in \Omega . \end{aligned}$$

The corresponding boundary conditions (BCs) are categorized in the following way:

- **Type 1:**
  7 $$\begin{aligned} \mathscr {U}(x_{0},t)&=\varepsilon _{0}(t),\quad \mathscr {U}(x_{N},t)=\delta _{0}(t), \quad t>0\nonumber \\ \mathscr {V}(x_{0},t)&=\varepsilon _{1}(t),\quad \mathscr {V}(x_{N},t)=\delta _{1}(t), \quad t>0. \end{aligned}$$
- **Type 2: **
  8 $$\begin{aligned} \mathscr {U}_{x}(x_{0},t)&=\varepsilon _{0}(t), \quad \mathscr {U}(x_{N},t)=\delta _{0}(t), \quad t>0\nonumber \\ \mathscr {V}_{x}(x_{0},t)&=\varepsilon _{1}(t), \quad \mathscr {V}(x_{N},t)=\delta _{1}(t), \quad t>0. \end{aligned}$$
- **Type 3: **
  9 $$\begin{aligned} \mathscr {U}_{x}(x_{0},t)&=\varepsilon _{0}(t),\quad \mathscr {U}_{x}(x_{N},t)=\delta _{0}(t), \quad t>0\nonumber \\ \mathscr {V}_{x}(x_{0},t)&=\varepsilon _{1}(t), \quad \mathscr {V}_{x}(x_{N},t)=\delta _{1}(t), \quad t>0. \end{aligned}$$

Now, based on the variation of the coefficient, Eq. (5) reduces to a variety of different linear and non-linear models. The models which will be under investigation, are listed in Table 1^38–41^:

**Table 1:** Coefficients of different RDMs.

Problems	$a_1$	$a_2$	$b_1$	$b_2$	$c_1$	$c_2$	$d_1$	$d_2$	$e_1$	$e_2$	$m_1$	$m_2$	$\kappa _1$	$\kappa _2$
Linear	*d*	*d*	$-a$	0	1	$-b$	0	0	0	0	0	0	0	0
Brusselator	$\varepsilon _1$	$\varepsilon _2$	-$\mathscr {B}$-1	$\mathscr {B}$	0	0	1	− 1	0	0	0	0	$\mathscr {A}$	0
Schnakenberg	1	*d*	$-\gamma$	0	0	0	$\gamma$	$-\gamma$	0	0	0	0	$\gamma a$	$\gamma b$
Gray-Scott	$\varepsilon _1$	$\varepsilon _2$	$-f$	0	0	$-f-k$	0	0	0	0	− 1	1	*f*	0

Using Eq. (2) and an implicit scheme to Eq. (5) the resultant is:

10 $$\begin{aligned}{} & {} \mathscr {A}_{\alpha } \sum _{k=0}^{n} \beta ^{\alpha }_k \left[ \left( \mathscr {U}\right) ^{n-k+1} -\left( \mathscr {U}\right) ^{n-k}\right] =a_{1} \left( \frac{\partial ^{2} \mathscr {U}}{\partial x^{2}}\right) ^{n+1} + b_{1} \left( \mathscr {U}\right) ^{n+1} + c_{1} \left( \mathscr {V}\right) ^{n+1} \nonumber \\{} & {} \quad +d_{1} \left( \mathscr {U}^{2} \mathscr {V}\right) ^{n+1} + e_{1} \left( \mathscr {U} \mathscr {V}\right) ^{n+1} + m_{1} \left( \mathscr {U} \mathscr {V}^{2}\right) ^{n+1} + \kappa _1 +\left( \mathscr {F}_{1}\right) ^{n+1}, \end{aligned}$$

11 $$\begin{aligned}{} & {} \mathscr {A}_{\alpha } \sum _{k=0}^{n} \beta ^{\alpha }_k \left[ \left( \mathscr {V}\right) ^{n-k+1} - \left( \mathscr {V}\right) ^{n-k}\right] =a_{2} \left( \frac{\partial ^{2} \mathscr {V}}{\partial x^{2}}\right) ^{n+1} + b_{2} \left( \mathscr {U}\right) ^{n+1} + c_{2} \left( \mathscr {V}\right) ^{n+1}\nonumber \\{} & {} \quad +d_{2} \left( \mathscr {U}^{2} \mathscr {V}\right) ^{n+1} + e_{2} \left( \mathscr {U} \mathscr {V}\right) ^{n+1} + m_{2} \left( \mathscr {U} \mathscr {V}^{2}\right) ^{n+1} + \kappa _2+\left( \mathscr {F}_{2}\right) ^{n+1}, \end{aligned}$$

where $$\left( \mathscr {U}\right) ^{n}=\mathscr {U}(x,t^n)$$, $$\left( \mathscr {V}\right) ^{n}=\mathscr {V}(x,t^n)$$, $$\left( \mathscr {F}_{1}\right) ^{n}=\mathscr {F}_{1}(x,t^{n})$$ and $$\left( \mathscr {F}_{2}\right) ^{n}=\mathscr {F}_{2}(x,t^{n})$$

Further simplification of the above Eqs. (10–11) leads to:

12 $$\begin{aligned}{} & {} a_{1} \left( \frac{\partial ^{2} \mathscr {U}}{\partial x^{2}}\right) ^{n+1} + b_{1} \left( \mathscr {U}\right) ^{n+1} + c_{1} \left( \mathscr {V}\right) ^{n+1} + d_{1} \left( \mathscr {U}^{2} \mathscr {V}\right) ^{n+1} + e_{1} \left( \mathscr {U} \mathscr {V}\right) ^{n+1} \nonumber \\{} & {} \quad +m_{1} \left( \mathscr {U} \mathscr {V}^{2}\right) ^{n+1} - \mathscr {A}_{\alpha } \left( \mathscr {U}\right) ^{n+1}= - \mathscr {A}_{\alpha } \left( \mathscr {U}\right) ^{n} - \left( \mathscr {F}_{1}\right) ^{n+1} - \kappa _1\nonumber \\{} & {} \quad +\mathscr {A}_{\alpha } \sum _{k=1}^{n} \beta ^{\alpha }_k \left[ \left( \mathscr {U}\right) ^{n-k+1} - \left( \mathscr {U}\right) ^{n-k}\right] , \end{aligned}$$

13 $$\begin{aligned}{} & {} a_{2} \left( \frac{\partial ^{2} \mathscr {V}}{\partial x^{2}}\right) ^{n+1} + b_{2} \left( \mathscr {U}\right) ^{n+1} + c_{2} \left( \mathscr {V}\right) ^{n+1} + d_{2} \left( \mathscr {U}^{2} \mathscr {V}\right) ^{n+1} + e_{2} \left( \mathscr {U} \mathscr {V}\right) ^{n+1} \nonumber \\{} & {} \quad +m_{2} \left( \mathscr {U} \mathscr {V}^{2}\right) ^{n+1} -\mathscr {A}_{\alpha } \left( \mathscr {V}\right) ^{n+1}=-\mathscr {A}_{\alpha } \left( \mathscr {V}\right) ^{n} - \kappa _2- \left( \mathscr {F}_{2}\right) ^{n+1} \nonumber \\{} & {} \quad +\mathscr {A}_{\alpha } \sum _{k=1}^{n} \beta ^{\alpha }_k \left[ \left( \mathscr {V}\right) ^{n-k+1} - \left( \mathscr {V}\right) ^{n-k}\right] . \end{aligned}$$

The nonlinear terms $\left( \mathscr {U}^{2} \mathscr {V}\right) ^{n+1}$, $\left( \mathscr {U} \mathscr {V}\right) ^{n+1}$, and $\left( \mathscr {U} \mathscr {V}^{2}\right) ^{n+1}$ are linearized using Qausilinearization^42^:

14 $$\begin{aligned} \left( \mathscr {U}^{2} \mathscr {V}\right) ^{n+1}= & {} 2 \left( \mathscr {U}\right) ^{n} \left( \mathscr {V}\right) ^{n} \left( \mathscr {U}\right) ^{n+1} -2 \left( \mathscr {U}^{2}\right) ^{n} \left( \mathscr {V}\right) ^{n} +\left( \mathscr {U}^{2}\right) ^{n} \left( \mathscr {V}\right) ^{n+1} \end{aligned}$$

15 $$\begin{aligned} \left( \mathscr{U}\mathscr{V}\right) ^{n+1}= & {} \left( \mathscr {V}\right) ^{n} \left( \mathscr {U}\right) ^{n+1} - \left( \mathscr{U}\mathscr{V}\right) ^{n} +\left( \mathscr {U}\right) ^{n} \left( \mathscr {V}\right) ^{n+1} \end{aligned}$$

16 $$\begin{aligned} \left( \mathscr{U}\mathscr{V}^{2}\right) ^{n+1}= & {} 2 \left( \mathscr {U}\right) ^{n} \left( \mathscr {V}\right) ^{n} \left( \mathscr {V}\right) ^{n+1} -2 \left( \mathscr {U}\right) ^{n} \left( \mathscr {V}^{2}\right) ^{n} +\left( \mathscr {V}^{2}\right) ^{n} \left( \mathscr {U}\right) ^{n+1} \end{aligned}$$

Inserting the central difference approximation of $\mathscr {U}_{xx}^{n+1}$ and $\mathscr {V}_{xx}^{n+1}$ together with non-linear terms Eqs. (14–16), in Eqs. (12) and (13) eventually gives linear system of equations with the compact form given by:

17 $$\begin{aligned} \mathscr {A}\mathscr {W}^{n+1}=\mathscr {W}^{n}, \end{aligned}$$

where the dimensions of the matrices of $\mathscr {A}$ and $\mathscr {W}$ are described below:

- For type 1 boundary conditions, the order of $\mathscr {A}$ and $\mathscr {W}$ will be ${(2\mathscr {M}-2)} \times {(2\mathscr {M}-2)}$ and $(2 \mathscr {M}-2)\times 1$, respectively.
- For type 2 boundary conditions, the order of $\mathscr {A}$ and $\mathscr {W}$ will be ${2\mathscr {M}} \times {2\mathscr {M}}$ and $2 \mathscr {M}\times 1$, respectively.
- For type 3 boundary conditions, the order of $\mathscr {A}$ and W will be ${(2\mathscr {M}+2)} \times {(2\mathscr {M}+2)}$ and $(2 \mathscr {M}+2)\times 1$, respectively.

## Stability analysis

In this part of the manuscript, the stability analysis of the numerical method for the TFRDMs model is discussed. Assume the coefficients for this model from the first row of Table 1, then the system reduces to:

18 $$\begin{aligned}{} & {} d\lambda \left( \mathscr {U}_{j-1}\right) ^{n+1} - (2d\lambda +a +\mathscr {A}_{\alpha }) \left( \mathscr {U}_{j}\right) ^{n+1}+ d\lambda \left( \mathscr {U}_{j+1}\right) ^{n+1} + \left( \mathscr {V}_{j}\right) ^{n+1} = - \mathscr {A}_{\alpha } \left( \mathscr {U}_{j}\right) ^{n} \nonumber \\{} & {} \quad +\mathscr {A}_{\alpha } \sum _{k=1}^{n} \beta ^{\alpha }_k \left[ \left( \mathscr {U}_{j}\right) ^{n-k+1} - \left( \mathscr {U}_{j}\right) ^{n-k}\right] \end{aligned}$$

19 $$\begin{aligned}{} & {} d\lambda \left( \mathscr {V}_{j-1}\right) ^{n+1} - (2d\lambda +b +\mathscr {A}_{\alpha }) \left( \mathscr {V}_{j}\right) ^{n+1}+ d\lambda \left( \mathscr {V}_{j+1}\right) ^{n+1} = - \mathscr {A}_{\alpha } \left( \mathscr {V}_{j}\right) ^{n} \nonumber \\{} & {} \quad +\mathscr {A}_{\alpha } \sum _{k=1}^{n} \beta ^{\alpha }_k \left[ \left( \mathscr {V}_{j}\right) ^{n-k+1} - \left( \mathscr {V}_{j}\right) ^{n-k}\right] . \end{aligned}$$

### Theorem

The implicit scheme (18-19), for the system (5), with $\alpha \in (0,1)$ on $x \in [x_{0}, x_{\mathbb {N}}]$ is unconditionally stable.

### Proof

Following the procedure^43^ we assume:

20 $$\begin{aligned} \left( \mathscr {U}_{j}\right) ^{n}= & {} \xi ^{n} e^{i\omega j h},\nonumber \\ \left( \mathscr {V}_{j}\right) ^{n}= & {} \eta ^{n} e^{i\omega j h}, \end{aligned}$$

where $i=\sqrt{-1}.$ Using Eq. (20) in Eqs. (18–19) and some algebraic manipulation leads to:

21 $$\begin{aligned} \xi ^{n+1}\left( \dfrac{2d\lambda }{\mathscr {A}_{\alpha }}(\cos (\omega h) -1) -\dfrac{a}{\mathscr {A}_{\alpha }} -1 \right) + \dfrac{ \eta ^{n+1}}{\mathscr {A}_{\alpha }}= & {} -\xi ^{n} + \sum _{k=1}^{n} \beta ^{\alpha }_k (\xi ^{n-k+1} - \xi ^{n-k} ), \end{aligned}$$

22 $$\begin{aligned} \eta ^{n+1}\left( \dfrac{2d\lambda }{\mathscr {A}_{\alpha }}(\cos (\omega h) -1) -\dfrac{b}{\mathscr {A}_{\alpha }} -1\right)= & {} -\eta ^{n} + \sum _{k=1}^{n} \beta ^{\alpha }_k (\eta ^{n-k+1} - \eta ^{n-k} ), \end{aligned}$$

From Eq. (22) we get:

23 $$\begin{aligned} \eta ^{n+1} = \frac{ \eta ^{n} + \sum _{k=1}^{n} \beta ^{\alpha }_k (\eta ^{n-k} - \eta ^{n-k+1} )}{\dfrac{2d\lambda }{\mathscr {A}_{\alpha }}(1-\cos (\omega h)) +\dfrac{b}{\mathscr {A}_{\alpha }} +1 }, \end{aligned}$$

Since $\dfrac{2d\lambda }{\mathscr {A}_{\alpha }}(1-\cos (\omega h) +\dfrac{b}{\mathscr {A}_{\alpha }} +1 \ge 1$, hence it follows:

24 $$\begin{aligned} \eta ^{n+1} \le { \eta ^{n} + \sum _{k=1}^{n} \beta ^{\alpha }_k (\eta ^{n-k} - \eta ^{n-k+1} )}. \end{aligned}$$

For $$n=0$$

25 $$\begin{aligned} \eta ^{1} \le \eta ^{0}. \end{aligned}$$

In a similar fashion the following hold:

$$\begin{aligned} \eta ^{2} \le \eta ^{1} + \beta ^{\alpha }_1 (\eta ^{0} - \eta ^{1} ). \end{aligned}$$

If two consecutive approximations are closed then their difference approaches zero hence:

$$\begin{aligned} \eta ^{2} \le \eta ^{1}. \end{aligned}$$

Continuing in this way one can write

26 $$\begin{aligned} \eta ^{n+1} \le \eta ^{n}\le \cdots \le \eta ^{0}. \end{aligned}$$

Now putting Eq. (23) in Eq. (21) we obtain:

27 $$\begin{aligned} \xi ^{n+1}\left( \dfrac{2d\lambda }{\mathscr {A}_{\alpha }}(\cos (\omega h) -1) -\dfrac{a}{\mathscr {A}_{\alpha }} -1 \right) + \dfrac{ \frac{ \eta ^{n} + \sum _{k=1}^{n} \beta ^{\alpha }_k (\eta ^{n-k} - \eta ^{n-k+1})}{\dfrac{2d\lambda }{\mathscr {A}_{\alpha }}(1-\cos (\omega h)) +\dfrac{b}{\mathscr {A}_{\alpha }} +1 }}{\mathscr {A}_{\alpha }} = -\xi ^{n} + \sum _{k=1}^{n} \beta ^{\alpha }_k (\xi ^{n-k+1} - \xi ^{n-k} ), \end{aligned}$$

which gives:

28 $$\begin{aligned} \xi ^{n+1} \le \dfrac{ \xi ^{n} + \sum _{k=1}^{n} \beta ^{\alpha }_k (\xi ^{n-k} - \xi ^{n-k+1} )}{\dfrac{2d\lambda }{\mathscr {A}_{\alpha }}(1-cos(\omega h)) +\dfrac{a}{\mathscr {A}_{\alpha }} +1}. \end{aligned}$$

From earlier discussion, the following result can be deduced:

29 $$\begin{aligned} \xi ^{n+1} \le \xi ^{n}\le \cdots \le \xi ^{0}. \end{aligned}$$

Thus, $\xi ^{n+1}= |\mathscr {U}_{j}^{n+1}| \le \xi ^{0}=|\mathscr {U}_{j}^{0}|=|f_{j}|$ and $\eta ^{n+1}= |\mathscr {V}_{j}^{n+1}| \le \xi ^{0}=|\mathscr {V}_{j}^{0}|=|f_{j}|$. These results imply that $||\mathscr {U}^{n}||_{l_{2}} \le ||f||_{l_{2}}$, $||\mathscr {V}^{n}||_{l_{2}} \le ||f||_{l_{2}}$ which is the stability condition.

## Algorithm

**Input**: $0<\alpha \le 1$, $a_{j} , b_{j} , c_{j} , d_{j}, e_{j} , m_{j}$ and $\kappa _j$ for j=1,2 and time step size $\tau$.

**Output**:Solve the system of PDEs numerically using FDM to evaluate approximate solutions $\mathscr {U}^n$ and $\mathscr {V}^n$.

**Step 1**: Generate computational grid over the domain $\Omega$, discretizing the spatial dimensions into a finite set of points.

**Step 2**: Apply central differences formula to discretize the spatial derivatives in the PDEs and quadrature formula for time derivative.

**Step 3**: Set n=0.

**Step 4**: Calculate matrices $\mathscr {A}$ and $\mathscr {W}^{n}$.

**Step 5**:Calculate $\mathscr {W}^{n+1}$ by using inversion method from $\mathscr {A}\mathscr {W}^{n+1}=\mathscr {W}^{n},$.

**Step 6**:Start time loop n=1:N

**Step 7**:Repeat the step 4-5.

## Numerical experiments

In this section, the proposed scheme is applied to some linear and non-linear RDMs. To assure the accuracy, global relative error (RE), $L_{2}$ and $L_\infty$ have been used which are defined below:

30 $$\begin{aligned} RE =\left( \frac{\sum _{j=1}^{N}|\chi _{j}^{n+1}-\chi _{j}^{n}|^{2}}{|\chi _{j}^{n+1}|} \right) ^{\frac{1}{2}}, \end{aligned}$$

where $\chi _{j}^{n+1}$ and $\chi _{j}^{n}$ are the approximate solutions at two consecutive time levels.

31 $$\begin{aligned} \mathbb {L}_{2}= & {} \left( \sum _{j=1}^{N}\left( \chi _{j}^{ext}-\chi _{j}^{app}\right) ^{2} \right) ^{\frac{1}{2}}, \end{aligned}$$

32 $$\begin{aligned} {\mathbb {L}}_{\infty }= & {} \max _{1\le j\le N} \left| \chi _{j}^{ext}-\chi _{j}^{app}\right| , \end{aligned}$$

where $\chi _{j}^{ext}$ and $\chi _{j}^{app}$ are the exact and approximate solutions, respectively.

### Problem 5.1 (linear model)

Consider the coefficients from the first row of Table 1 the resultant equation is given by^38–41^:

33 $$\begin{aligned} \frac{\partial ^{\alpha } \mathscr {U}(x,t)}{\partial t^{\alpha }}&=d \frac{\partial ^{2} \mathscr {U}(x,t)}{\partial x^{2}} -a \mathscr {U}(x,t)+\mathscr {V}(x,t) + \mathscr {F}_{1}\nonumber \\ \frac{\partial ^{\alpha } \mathscr {V}(x,t)}{\partial t^{\alpha }}&=d \frac{\partial ^{2} \mathscr {V}(x,t)}{\partial x^{2}}-b \mathscr {V} (x,t) + \mathscr {F}_{2}. \end{aligned}$$

The corresponding ICs and BCs are:

34 $$\begin{aligned} \mathscr {U}(x,0)= & {} 2 \cos (x),\quad 0\le x \le \pi /2,\nonumber \\ \mathscr {V}(x,0)= & {} (a-b) \cos (x),\quad 0\le x \le \pi /2, \end{aligned}$$

35 $$\begin{aligned} \mathscr {U}_{x}(0,t)= & {} 0, \ \ \mathscr {U}(\pi /2,t) =0, \quad 0<t\le 1, \nonumber \\ \mathscr {V}_{x}(0,t)= & {} 0, \ \ \mathscr {V}(\pi /2,t) =0, \quad 0<t\le 1. \end{aligned}$$

The closed-form solutions of this system are:

$$\begin{aligned} \mathscr {U}(x,t)= & {} \left( e^{-(a+d)t}+e^{-(b+d)t}\right) \cos x,\\ \mathscr {V}(x,t)= & {} (a-b)\left( e^{-(b+d)t}\right) \cos x. \end{aligned}$$

Using the fact ($~^{\mathscr {C}}_{0}\mathscr {D}_{x}^{\alpha }(e^{\lambda x}) =\lambda ^{n} x^{n-\alpha } \mathbb {E}_{1,n-\alpha +1}(\lambda x)$) the associated source terms are extracted as follows:

$$\begin{aligned}{} & {} \mathscr {F}_{1}=-cos(x)t^{1-\alpha }[(a+d)E_{1,2-\alpha } (-(a+d)t)+(b+d)E_{1,2-\alpha }(-(b+d)t)]\\{} & {} \quad +cos(x)[(a+d)e^{-(a+d)t}+(b+d)e^{-(b+d)t}]\\{} & {} \mathscr {F}_{2}=-(a-b)(b+d)cos(x)t^{1-\alpha }(a+d) E_{1,2-\alpha }(-(b+d)t)\\{} & {} \quad +(a-b)(b+d)cos(x)e^{-(b+d)t}. \end{aligned}$$

For numerical simulations three cases are addressed here.

#### Diffusion-dominated

For diffusion-dominated case the parameters considered are a = 0.1, b = 0.01 and d = 1.

#### Reaction-dominated

For the reaction-dominated case the parameters considered are a = 2, b = 1 and d = 0.001

#### Reaction-dominated with stiff reaction

For this case the selection of parameters are a = 100, b = 1 and d = 0.001.

**Figure 1:**
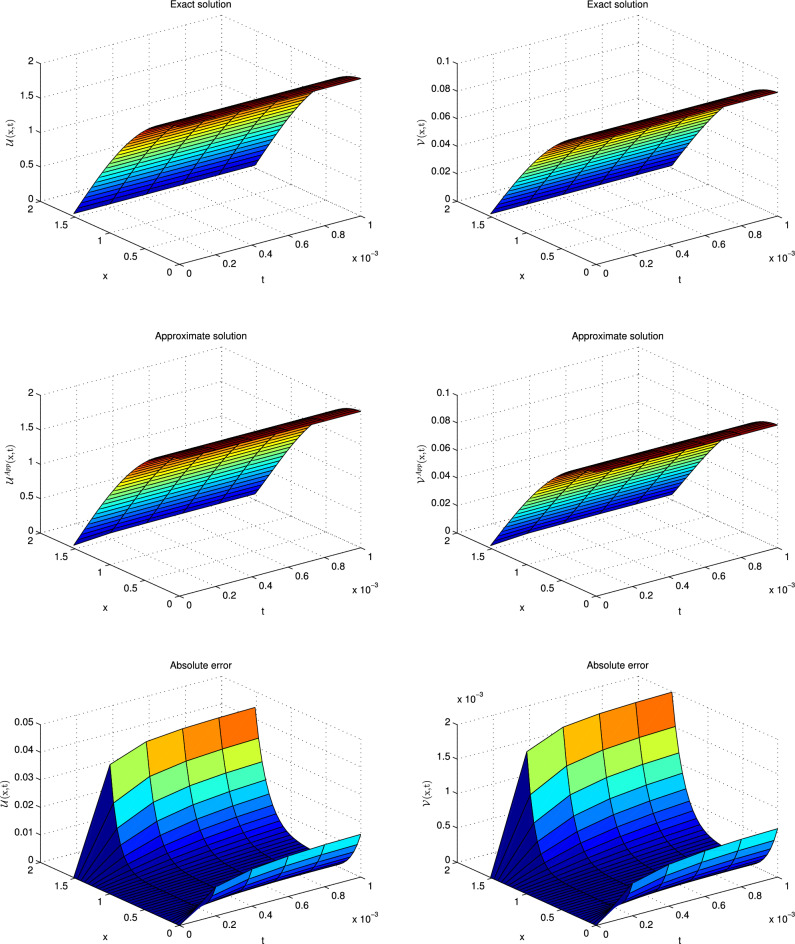
Solution profile with absolute error of $\mathscr {U}$ and $\mathscr {V}$ at $t=0.001$ for diffusion dominant case.

**Figure 2:**
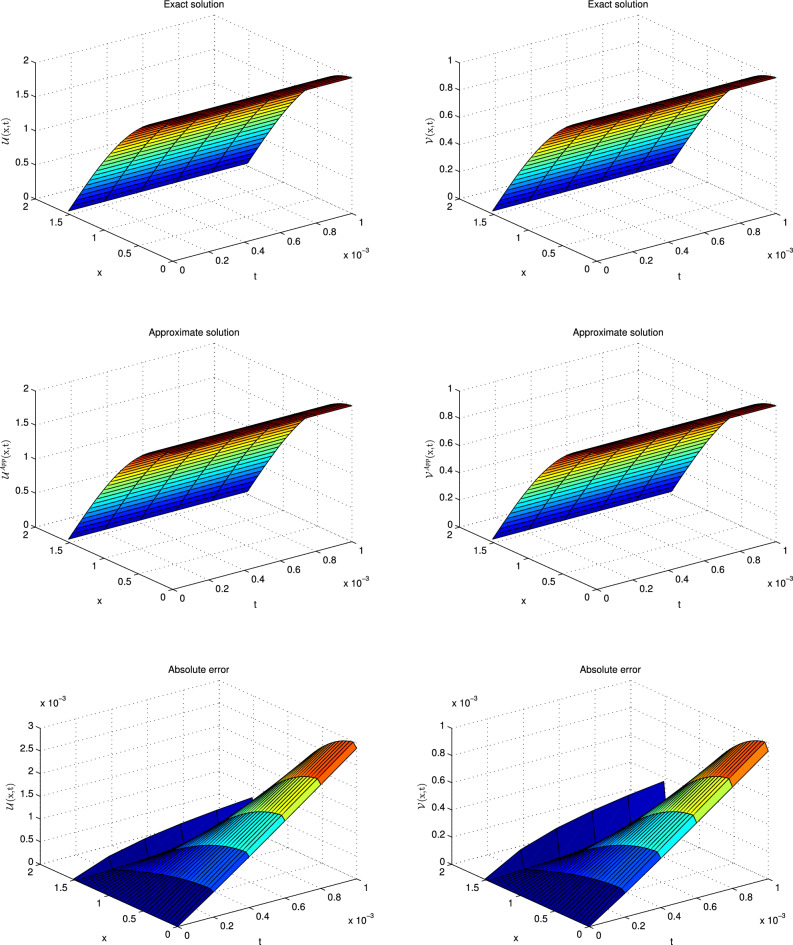
Solutions profile of $\mathscr {U}$ and $\mathscr {V}$ at $t=0.001$ for reaction dominant case.

**Figure 3:**
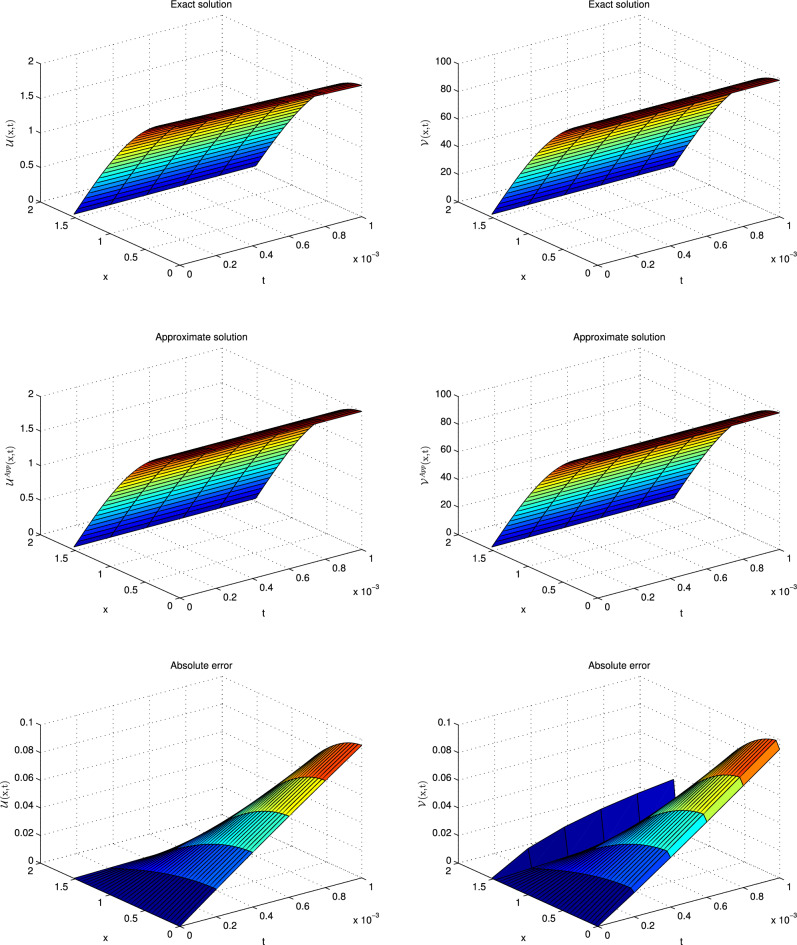
Solutions profile of $\mathscr {U}$ and $\mathscr {V}$ at $t=0.001$ for reaction dominant with stiff reaction.

This problem has been solved numerically, with the suggested technique, and the obtained results are noted in the form of tabulated and graphical forms. In Tables 2, 3, 4, 5, 6 and 7, the $L_{2}$ and $L_{\infty }$ error norms are recorded for different times and $\alpha$. The consecutive Tables 2 and 3 show the results for diffusion-dominated case, Tables 4 and 5 for reaction-dominated case and Tables 6 and 7 for reaction-dominated with stiff reaction case, respectively. Tabulated simulations reveal the good performance of the present technique. Similarly, solutions profile of exact versus numerical solutions are displayed in Figs. 1, 2 and 3 for diffusion dominated, reaction dominated and reaction dominated with stiff reaction using $\alpha =0.5$. From the graphical results it is plainly visible, that both solutions are in good agreement.

**Table 2:** $\mathbb {L}_\infty$ norms for distinct values of $\alpha$ at different time levels in the diffusion-dominated when a = 0.1, b = 0.01, d = 1.

t	$\alpha =0.25$		$\alpha =0.5$		$\alpha =0.75$		$\alpha =1$	
	$\mathbb {L}_{\infty }$ of $\mathscr {U}$	$\mathbb {L}_{\infty }$ of $\mathscr {V}$	$\mathbb {L}_{\infty }$ of $\mathscr {U}$	$\mathbb {L}_{\infty }$ of $\mathscr {V}$	$\mathbb {L}_{\infty }$ of $\mathscr {U}$	$\mathbb {L}_{\infty }$ of $\mathscr {V}$	$\mathbb {L}_{\infty }$ of $\mathscr {U}$	$\mathbb {L}_{\infty }$ of $\mathscr {V}$
0.001	6.0820e−03	2.7371e−04	6.0245e−03	2.7112e−04	5.9195e−03	2.6639e−04	5.7664e−03	2.5950e−04
0.01	6.0349e−03	2.7170e−04	6.0126e−03	2.7069e−04	5.9832e−03	2.6937e−04	5.9586e−03	2.6826e−04
0.05	5.7908e−03	2.6118e−04	5.7825e−03	2.6080e−04	5.7755e−03	2.6048e−04	5.7753e−03	2.6047e−04
0.075	5.6416e−03	2.5473e−04	5.6360e−03	2.5448e−04	5.6328e−03	2.5433e−04	5.6321e−03	2.5447e−04
0.1	5.4960e−03	2.4844e−04	5.4924e−03	2.4827e−04	5.4916e−03	2.4823e−04	5.4914e−03	2.4818e−04

**Table 3:** $\mathbb {L}_{2}$ norms for distinct values of $\alpha$ at different time levels in the diffusion-dominated when a = 0.1, b = 0.01, d = 1.

t	$\alpha =0.25$		$\alpha =0.5$		$\alpha =0.75$		$\alpha =1$	
	$\mathbb {L}_{2}$ of $\mathscr {U}$	$\mathbb {L}_{2}$ of $\mathscr {V}$	$\mathbb {L}_{2}$ of $\mathscr {U}$	$\mathbb {L}_{2}$ of $\mathscr {V}$	$\mathbb {L}_{2}$ of $\mathscr {U}$	$\mathbb {L}_{2}$ of $\mathscr {V}$	$\mathbb {L}_{2}$ of $\mathscr {U}$	$\mathbb {L}_{2}$ of $\mathscr {V}$
0.001	5.0461e−02	2.2745e−03	3.3379e−02	1.5027e−03	2.2302e−02	1.0037e−03	1.4967e−02	6.7357e−04
0.01	5.5703e−02	2.5131e−03	4.4871e−02	2.0224e−03	3.4863e−02	1.5700e−03	2.7260e−02	1.2273e−03
0.05	5.6607e−02	2.5591e−03	5.1516e−02	2.3267e−03	4.6751e−02	2.1082e−03	4.1236e−02	1.8538e−03
0.075	5.5951e−02	2.5315e−03	5.2535e−02	2.3720e−03	5.0255e−02	2.2612e−03	4.7814e−02	2.1335e−03
0.1	5.5298e−02	2.5025e−03	5.3686e−02	2.4178e−03	5.3054e−02	2.4174e−03	5.2420e−02	2.2854e−03

**Table 4:** $\mathbb {L}_\infty$ norms for distinct values of $\alpha$ at different time levels in the reaction-dominated when when a = 2, b = 1, d = 0.001.

t	$\alpha =0.25$		$\alpha =0.5$		$\alpha =0.75$		$\alpha =1$	
	$\mathbb {L}_{\infty }$ of $\mathscr {U}$	$\mathbb {L}_{\infty }$ of $\mathscr {V}$	$\mathbb {L}_{\infty }$ of $\mathscr {U}$	$\mathbb {L}_{\infty }$ of $\mathscr {V}$	$\mathbb {L}_{\infty }$ of $\mathscr {U}$	$\mathbb {L}_{\infty }$ of $\mathscr {V}$	$\mathbb {L}_{\infty }$ of $\mathscr {U}$	$\mathbb {L}_{\infty }$ of $\mathscr {V}$
0.001	4.6895e−03	2.3665e−03	3.5473e−03	1.7817e−03	1.9870e−03	9.9530e−04	5.7697e−04	2.8865e−04
0.01	4.8733e−03	2.4754e−03	4.3649e−03	2.2087e−03	3.7403e−03	1.8860e−03	2.8101e−03	1.4127e−03
0.05	4.7159e−03	2.4461e−03	4.5045e−03	2.3300e−03	4.3338e−03	2.2334e−03	4.2020e−03	2.1545e−03
0.075	4.5710e−03	2.4002e−03	4.4164e−03	2.3132e−03	4.3221e−03	2.2553e−03	4.3050e−03	2.2335e−03
0.1	4.4232e−03	2.3505e−03	4.3049e−03	2.2822e−03	4.2558e−03	2.2476e−03	4.3001e−03	2.2567e−03

**Table 5:** $\mathbb {L}_2$ norms for distinct values of $\alpha$ at different time levels in the reaction-dominated when when a = 2, b = 1, d = 0.001.

t	$\alpha =0.25$		$\alpha =0.5$		$\alpha =0.75$		$\alpha =1$	
	$\mathbb {L}_{2}$ of $\mathscr {U}$	$\mathbb {L}_{2}$ of $\mathscr {V}$	$\mathbb {L}_{2}$ of $\mathscr {U}$	$\mathbb {L}_{2}$ of $\mathscr {V}$	$\mathbb {L}_{2}$ of $\mathscr {U}$	$\mathbb {L}_{2}$ of $\mathscr {V}$	$\mathbb {L}_{2}$ of $\mathscr {U}$	$\mathbb {L}_{2}$ of $\mathscr {V}$
0.001	7.3047e−03	3.7330e−03	4.3563e−03	2.1923e−03	2.0693e−03	1.0366e−03	5.7805e−04	2.8918e−04
0.01	8.2597e−03	4.2811e−03	6.3431e−03	3.2291e−03	4.6695e−03	2.3454e−03	3.1145e−03	1.5238e−03
0.05	9.2742e−03	4.5919e−03	1.0909e−02	4.2832e−03	1.3506e−02	4.2313e−03	1.8929e−02	4.7582e−03
0.075	1.1636e−02	4.9199e−03	1.8456e−02	5.5718e−03	2.5948e−02	6.5884e−03	3.9181e−02	8.8837e−03
0.1	1.6020e−02	5.6583e−03	2.9267e−02	7.7966e−03	4.2602e−02	1.0189e−02	6.5769e−02	1.4717e−02

**Table 6:** $\mathbb {L}_\infty$ norms for distinct values of $\alpha$ at different time levels in the reaction-dominated with stiff when when a = 100, b = 1, d = 0.001.

t	$\alpha =0.25$		$\alpha =0.5$		$\alpha =0.75$		$\alpha =1$	
	$\mathbb {L}_{\infty }$ of $\mathscr {U}$	$\mathbb {L}_{\infty }$ of $\mathscr {V}$	$\mathbb {L}_{\infty }$ of $\mathscr {U}$	$\mathbb {L}_{\infty }$ of $\mathscr {V}$	$\mathbb {L}_{\infty }$ of $\mathscr {U}$	$\mathbb {L}_{\infty }$ of $\mathscr {V}$	$\mathbb {L}_{\infty }$ of $\mathscr {U}$	$\mathbb {L}_{\infty }$ of $\mathscr {V}$
0.001	3.2176e−03	2.9633e−01	8.6635e−04	6.7405e−02	6.0743e−04	1.1838e−02	1.4373e−04	2.0600e−03
0.01	4.3451e−03	4.1886e−01	2.6476e−03	1.8405e−01	1.5799e−03	6.2873e−02	8.0842e−04	2.0158e−02
0.05	5.0192e−03	4.9547e−01	4.5914e−03	3.4635e−01	3.4836e−03	2.0612e−01	2.8933e−03	1.0922e−01
0.075	5.1232e−03	5.0579e−01	4.6426e−03	3.8353e−01	4.1662e−03	2.5651e−01	2.5435e−03	1.5425e−01
0.1	5.2140e−03	5.1566e−01	5.0360e−03	3.8781e−01	4.2630e−03	2.6075e−01	4.1022e−03	1.6462e−01

**Table 7:** $\mathbb {L}_2$ norms for distinct values of $\alpha$ at different time levels in the reaction-dominated with stiff when when a = 100, b = 1, d = 0.001.

t	$\alpha =0.25$		$\alpha =0.5$		$\alpha =0.75$		$\alpha =1$	
	$\mathbb {L}_{2}$ of $\mathscr {U}$	$\mathbb {L}_{2}$ of $\mathscr {V}$	$\mathbb {L}_{2}$ of $\mathscr {U}$	$\mathbb {L}_{2}$ of $\mathscr {V}$	$\mathbb {L}_{2}$ of $\mathscr {U}$	$\mathbb {L}_{2}$ of $\mathscr {V}$	$\mathbb {L}_{2}$ of $\mathscr {U}$	$\mathbb {L}_{2}$ of $\mathscr {V}$
0.001	3.2467e−03	2.9931e−01	1.0402e−03	6.7622e−02	3.0531e−03	1.1873e−02	7.2142e−04	2.0664e−03
0.01	4.6633e−03	4.2629e−01	4.3442e−03	1.8500e−01	4.0301e−03	6.3150e−02	8.506e−04	2.1582e−02
0.05	1.1079e−02	5.0874e−01	1.0922e−02	3.5317e−01	6.8222e−03	2.2716e−01	8.4562e−03	2.0929e−01
0.075	1.2536e−02	5.2131e−01	1.1626e−02	4.0113e−01	1.0644e−02	3.2946e−01	9.2769e−03	2.1055e−01
0.1	6.9750e−02	5.3264e−01	1.6586e−02	4.2573e−01	1.3483e−02	4.5355e−01	1.0593e−02	7.3185e−01

### Problem 5.2 (Brusselator model)

The exact solution this model and the next three nonlinear models is not available. For these cases ignored the corresponding source term. The numerical results in non-fractional form are given in literature. We tried the proposed technique for different values of $\alpha$ and observed its graphical behavior which approaches towards the integer value of $\alpha =1$. In these cases the relative error is measured between two consecutive time levels. On behalf of these arguments we claim that proposed method works for such nonlinear fractional models. Using the coefficients from the second row of Table 1, which gives the following Brusselator model of kinetics for two chemical components^38–41^:

36 $$\begin{aligned} \frac{\partial ^{\alpha } \mathscr {U}(x,t)}{\partial t^{\alpha }}&=\varepsilon _{1} \mathscr {U}_{xx} - \left( \mathscr {B} +1\right) \mathscr {U} + \mathscr {U}^{2} \mathscr {V} + \mathscr {A} ,\nonumber \\ \frac{\partial ^{\alpha } \mathscr {V}(x,t)}{\partial t^{\alpha }}&=\varepsilon _{2} \mathscr {V}_{xx} + \mathscr {B} \mathscr {U} - \mathscr {U}^{2} \mathscr {V}, \end{aligned}$$

with ICs:

37 $$\begin{aligned} \mathscr {U}(x,0)&=0.5, \nonumber \\ \mathscr {V}(x,0)&=1+5x+\frac{1}{4}{\tanh (20x)} -\frac{1}{4}{\tanh (20(x-1))}, \end{aligned}$$

and the BCs are:

38 $$\begin{aligned} \mathscr {U}_x(0,t)&=0,\quad \mathscr {U}(1,t)=0, \nonumber \\ \mathscr {V}_x(0,t)&=0,\quad \mathscr {V}(1,t)=0. \end{aligned}$$

**Figure 4:**
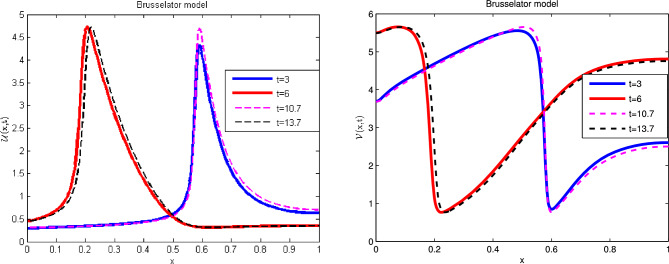
Numerical illustration of Brusselator model for $\mathscr {U}$ and $\mathscr {V}$ when $\alpha =1.$.

**Figure 5:**
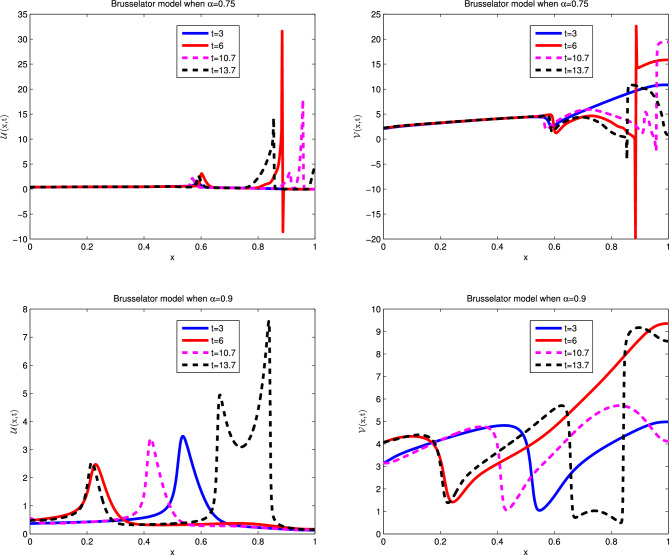
Numerical illustration of Brusselator model for $\mathscr {U}$ and $\mathscr {V}$ when $\alpha =0.75$ and $\alpha =0.9$.

Simulations are performed for the parameters: $\varepsilon _1 =\varepsilon _2 = 0.00002, {\mathscr {A}} = 1, {\mathscr {B}} = 3.4$, used in^41^ over the problem domain [0,1] . The relative error values for ${\mathscr {U}}$ and ${\mathscr {V}}$ at various values of $\alpha$ and different time levels are presented in Table 8. Similarly, the density values for periodical motion are given in Table 9. Tabulated data discloses that the proposed technique gives good results in terms of error values which are comparable with the previous results in literature. Graphically, the numerical solutions for integer derivative and times $t=3, 6, 10.7,13.7$ are shown in Fig. 4, which predict the oscillatory behavior in chemical reactions. In Fig. 5 the results are calculated for various values of fractional derivative, which retain the structure of oscillations, only the peaks are different due to fractional values of $\alpha$. From a graphical view two things are clear, the results agree with classical solutions and the scheme does not alter the physical meaning of the model with fractional derivative.

**Table 8:** Relative error for various $\alpha$ values at various time levels when $dt=0.01$ and $dx=0.05$ of the Brusselator model.

t	$\alpha =0.25$		$\alpha =0.5$		$\alpha =0.75$		$\alpha =1$	
	*RE* of $\mathscr {U}$	*RE* of $\mathscr {V}$	*RE* of $\mathscr {U}$	*RE* of $\mathscr {V}$	*RE* of $\mathscr {U}$	*RE* of $\mathscr {V}$	*RE* of $\mathscr {U}$	*RE* of $\mathscr {V}$
3	2.0354e−10	2.2025e−10	3.7863e−04	3.5430e−03	1.5726e−02	7.0784e−03	1.7728e−02	7.0979e−03
6	9.9154e−15	4.4068e−15	2.8500e−04	1.6795e−03	2.2394e−02	3.7622e−03	3.4562e−04	8.2614e−04
10	7.5003e−16	2.0311e−15	2.1909e−02	6.8632e−03	2.1484e−02	1.9623e−03	1.9945e−02	7.7037e−03
13	6.0902e−15	6.8191e−16	4.1097e−04	3.7381e−03	6.2843e−03	1.6586e−03	1.0856e−02	4.6599e−03

**Table 9:** Density value for periodic motion of Brusselator model.

Density	*x*	0	0.2	0.4	0.6	0.8	1
U	3	0.294851285042341	0.342874476982005	0.439887206799160	4.084153286206512	0.868264657607814	0.631669227084894
	6	0.455133646517029	4.637328613106386	1.362737460708937	0.316810767726646	0.341809435938578	0.353024226227808
	10.7	0.315507295348274	0.340444936787278	0.426904326384855	4.422181773179734	0.975057358624334	0.703916805055259
	13.7	0.453599334757512	3.647001834026506	1.500682343948168	0.319195589894421	0.338228804864134	0.348914444829436
V	3	3.687773918047040	4.682788687309119	5.410800581895748	0.844858452761222	2.299626799142092	2.604506569694734
	6	5.499253473873485	1.150072687778154	1.840424829740125	3.662292719056844	4.649894995801780	4.809935414837560
	10.7	3.680063713497682	4.627752556692187	5.392449423645793	0.770900658236533	2.184852828836223	2.501560571021531
	13.7	5.491790039896567	2.426212381897124	1.738222712948466	3.605397600513641	4.591809581911599	4.754792117638447

### Problem 5.3 (Gray–Scott model)

Here, we take the values of coefficients from the third row of Table 1 which gives the Gray-Scot model^38–41^

39 $$\begin{aligned} \frac{\partial ^{\alpha } \mathscr {U}(x,t)}{\partial t^{\alpha }}&=\varepsilon _{1} \mathscr {U}_{xx} - \mathscr{U}\mathscr{V}^2 + f\left( 1-\mathscr {U}\right) ,\nonumber \\ \frac{\partial ^{\alpha } \mathscr {V}(x,t)}{\partial t^{\alpha }}&=\varepsilon _{2} \mathscr {V}_{xx}+ \mathscr{U}\mathscr{V}^2 -\left( f+k\right) \mathscr {V}. \end{aligned}$$

The ICs are:

40 $$\begin{aligned} \mathscr {U}(x,0)&=1-\frac{1}{2} {\sin ^{100} \left( \pi \frac{x-\mathscr {L}}{2\mathscr {L}}\right) }\nonumber \\ \mathscr {V}(x,0)&=\frac{1}{4} {\sin ^{100} \left( \pi \frac{x-\mathscr {L}}{2\mathscr {L}}\right) , } \end{aligned}$$

and the Dirichlet type of BCs are given below:

41 $$\begin{aligned} \mathscr {U}(-\mathscr {L},t)&=1,\quad \mathscr {U}(\mathscr {L},t)=1\nonumber \\ \mathscr {V}(-\mathscr {L},t)&=1,\quad \mathscr {V}(\mathscr {L},t)=1. \end{aligned}$$

**Figure 6:**
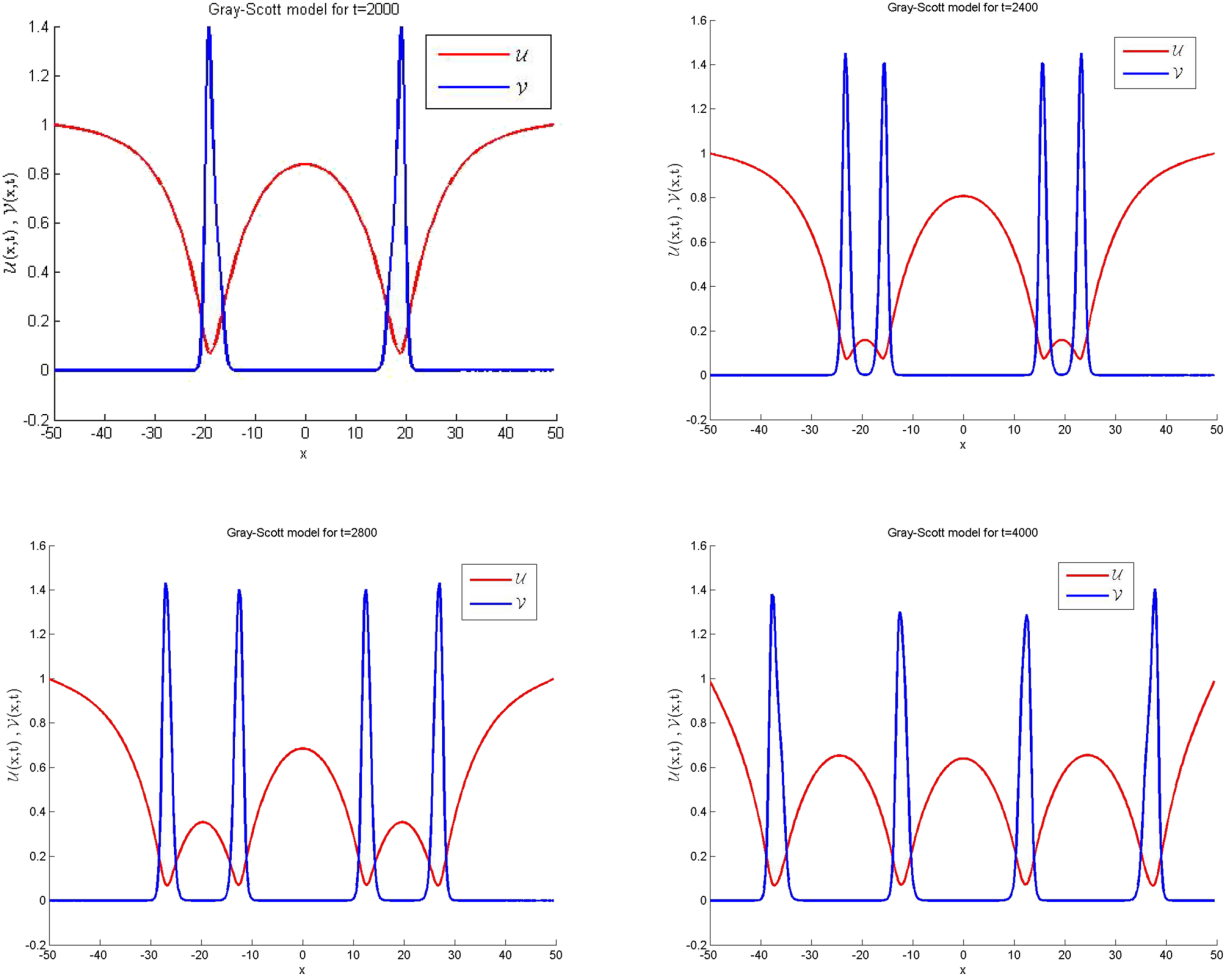
Numerical illustration for Gray–Scott model for $\mathscr {U}$ and $\mathscr {V}$ when $\alpha =1.$.

**Figure 7:**
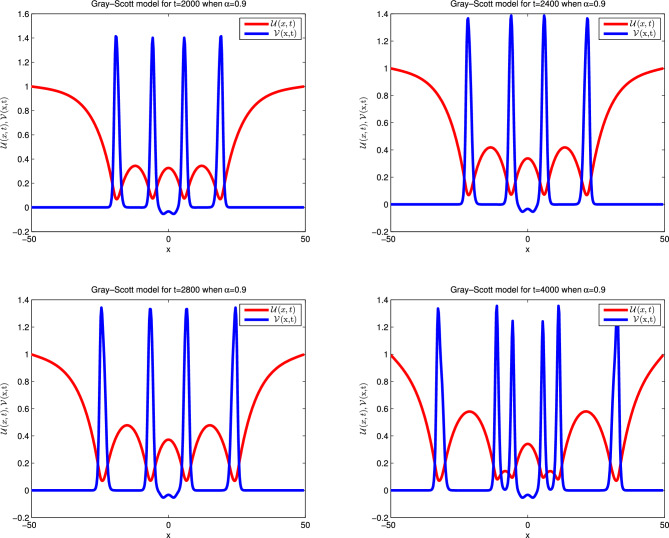
Numerical illustration for Gray–Scott model in 2D Graph when $dt=0.5$, $dx=0.25$ $\alpha =0.9$.

**Figure 8:**
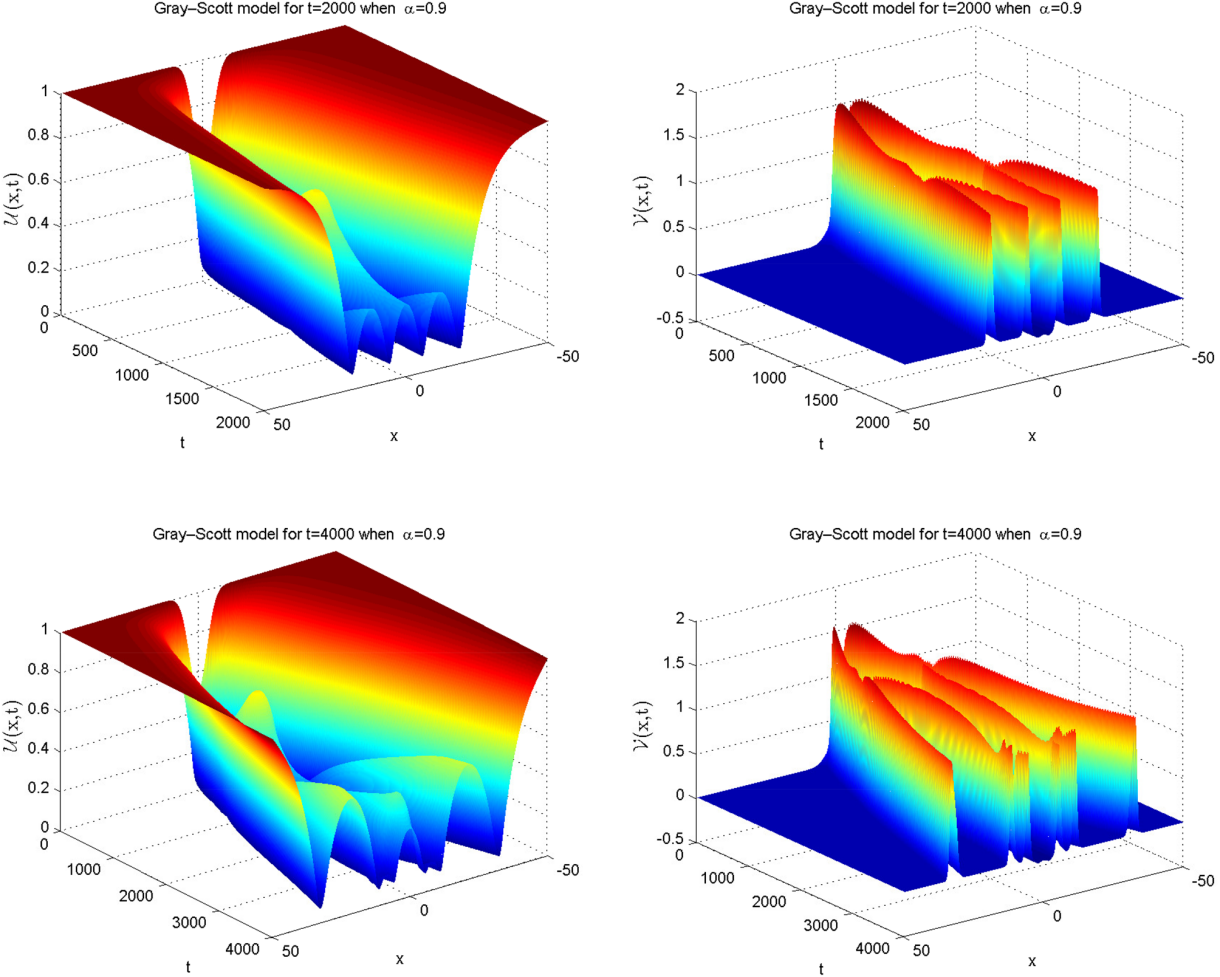
Numerical illustration of Gray–Scott model in 3D Graph when $dt=0.5$, $dx=0.25$ $\alpha =0.9.$.

Simulations are carried out for the spatial domain $[-\mathscr {L} , \mathscr {L}]$, with parameter values $\mathscr {L}=50$, $\varepsilon _1 = 1, \varepsilon _2 = 0.01$, $f = 0.02$, $k = 0.066$, which are taken from^41^. The achieved error values for differena longues of $\alpha$ and time are presented in Table 10. Tabulated simulation reveals that the scheme works well for large time. Numerical solutions are plotted in Fig. 6 for integer cases which show that the outcome is in good agreement with available results in previous work. Likewise, in Fig. 7 the simulations are noted for fractional cases which indicate that the graphical behavior is nearly similar to integer when $\alpha =0.9$. In Fig. 8 three dimensions numerical solutions are shown which show the wave type pattern.

**Table 10:** Relative error for various $\alpha$ values at various time levels when $dt=100$ and $dx=1$ of the Gray-Scott model.

t	$\alpha =0.25$		$\alpha =0.5$		$\alpha =0.75$		$\alpha =1$	
	*RE* of $\mathscr {U}$	*RE* of $\mathscr {V}$	*RE* of $\mathscr {U}$	*RE* of $\mathscr {V}$	*RE* of $\mathscr {U}$	*RE* of $\mathscr {V}$	*RE* of $\mathscr {U}$	*RE* of $\mathscr {V}$
2000	1.5586e−03	2.4029e−03	2.5015e−03	1.7205e−03	5.3181e−07	1.3595e−06	1.7787e−09	1.3074e−09
2400	2.2024e−04	1.5784e−04	6.6420e−04	3.6827e−04	7.6539e−08	1.2872e−07	2.6013e−11	1.9011e−11
2800	3.8928e−05	2.7964e−05	1.9440e−04	7.4461e−05	1.1049e−08	1.2020e−08	3.3840e−13	4.1099e−13
4000	8.5794e−07	1.7319e−06	6.2215e−06	1.2775e−06	3.3900e−11	7.6449e−12	1.0078e−13	1.0261e−13

### Problem 5.4 (Schnakenberg model)

Choosing the coefficient values from the fourth row of Table 1, we obtain the following Schnakenberg model^38–41^:

42 $$\begin{aligned} \frac{\partial ^{\alpha } \mathscr {U}(x,t)}{\partial t^{\alpha }}&=d_{1} \mathscr {U}_{xx} + \gamma \left( a_1 - \mathscr {U} +\mathscr {U}^{2} \mathscr {V}\right) ,\nonumber \\ \frac{\partial ^{\alpha } \mathscr {V}(x,t)}{\partial t^{\alpha }}&=d_{2} \mathscr {V}_{xx} + \gamma \left( b_1 - \mathscr {U}^{2} \mathscr {V}\right) , \end{aligned}$$

where ${\mathscr {U}}$ and ${\mathscr {V}}$ denote the activator and inhibitor concentrations, respectively, and *d* is diffusion coefficients, $\gamma$, *a*, and *b* are biological reaction rate constants. The following are the associated ICs:

43 $$\begin{aligned} \mathscr {U}(x,0)&=0.919145+{\frac{1}{1000}}{\sum _{j=1}^{25} {\frac{\cos (2\pi jx)}{j}}}\nonumber \\ \mathscr {V}(x,0)&=0.937903+ {\frac{1}{1000}}{\sum _{j=1}^{25} {\frac{\cos (2\pi jx)}{j}}}, \end{aligned}$$

and the BCs are:

44 $$\begin{aligned} \mathscr {U}_x(0,t)&=0,\quad \mathscr {U}_{x}(1,t)=0\nonumber \\ \mathscr {V}_x(0,t)&=0,\quad \mathscr {V}_{x}(1,t)=0. \end{aligned}$$

**Figure 9:**
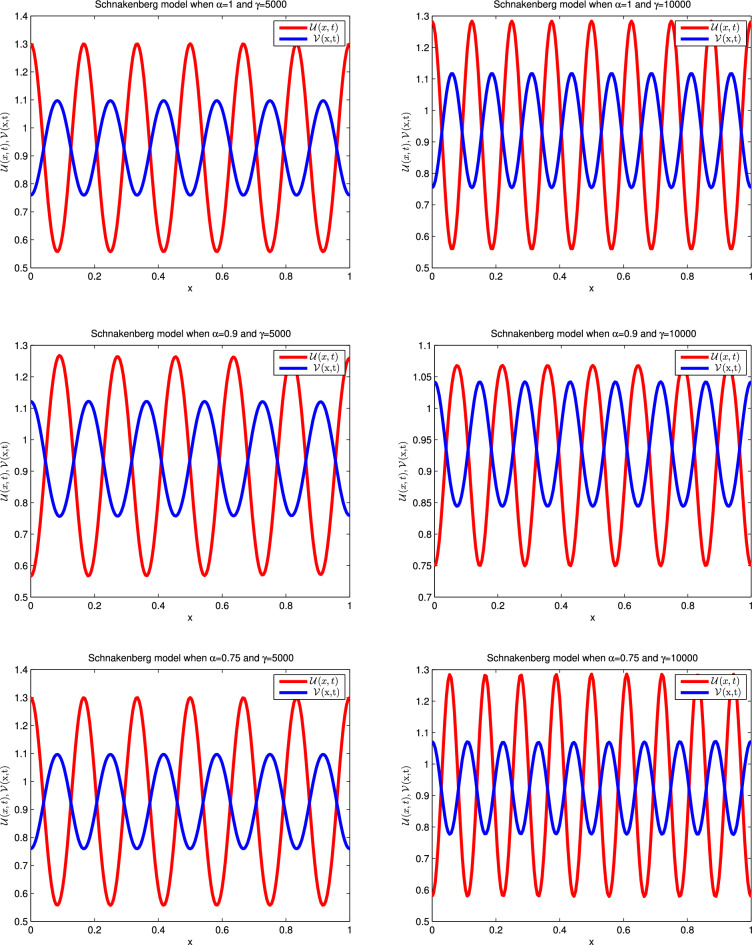
Numerical illustration for Schnakenberg model in one dimensional graph for $\mathscr {U}$ and $\mathscr {V}$ for $\gamma =5000$ and $\gamma =10{,}000$ at t=2.5 when $dt=0.001$, $dx=0.005$ and $\alpha =0.75,0.9$ and 1.

**Figure 10:**
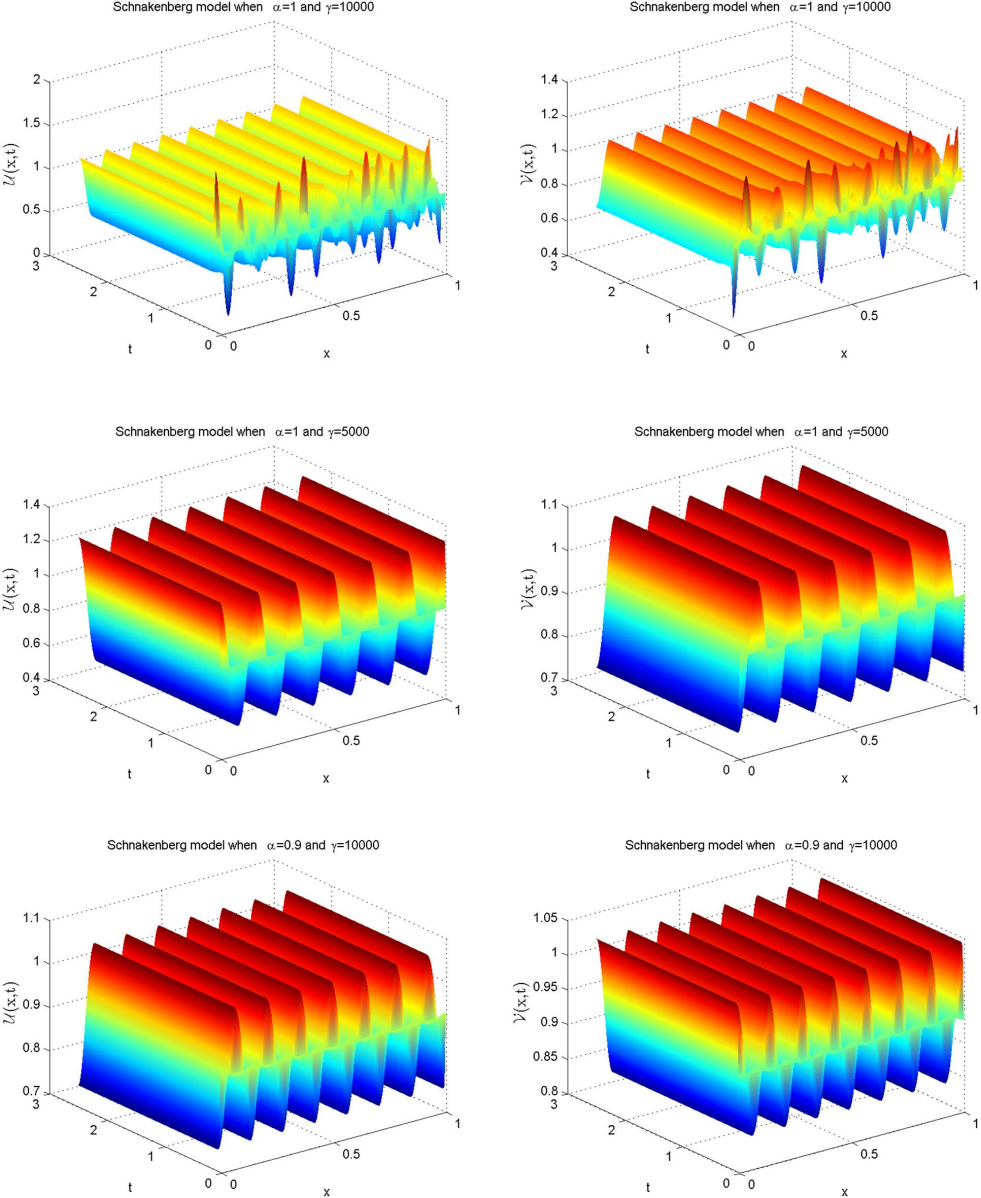
Numerical illustration of the Schnakenberg model in 3D graph for $\mathscr {U}$ and $\mathscr {V}$ for $\gamma =5000$ and $\gamma =10{,}000$ at t=2.5 when $dt=0.001$, $dx=0.005$ and $\alpha =0.9$ and 1.

To solve this model, we use $a = 0.126779, b = 0.792366, d = 10$, and $\gamma = 5000, 10000$. The computed simulations in the form of relative error are noted in Table 11. From tabulated values, it is obvious that computed solutions are pretty much accurate. The one-dimensional solution profiles are plotted in Fig. 9 for integer and fractional values of $\alpha$ which provide a clear picture of oscillatory motion. Moreover, the solutions in the three-dimensional form are presented in Fig. 10.

**Table 11:** Relative error for various $\alpha$ values at various time levels when $t=2.5$ and $dx=0.01$ of the Schnakenberg model when $\gamma =10{,}000$.

dt	$\alpha =0.25$		$\alpha =0.5$		$\alpha =0.75$		$\alpha =1$	
	*RE* of $\mathscr {U}$	*RE* of $\mathscr {V}$	*RE* of $\mathscr {U}$	*RE* of $\mathscr {V}$	*RE* of $\mathscr {U}$	*RE* of $\mathscr {V}$	*RE* of $\mathscr {U}$	*RE* of $\mathscr {V}$
0.1	5.1715e−15	4.7112e−15	1.4203e−15	1.1778e−15	2.3672e−15	2.3556e−15	2.5858e−15	6.5671e−15
0.01	4.0789e−15	3.4977e−15	2.9864e−15	3.7118e−15	3.0592e−15	2.1771e−15	4.9530e−15	7.2095e−15
0.005	2.4401e−15	5.8533e−15	4.0061e−15	2.6768e−15	4.2246e−15	1.5347e−15	3.7512e−15	2.1414e−15
0.001	3.1685e−15	1.6061e−15	1.9302e−15	1.3661e−15	4.5524e−15	6.7813e−15	3.1685e−15	5.1038e−15

### Ethics approval and consent to participate

In this work, no materials of any other person are used. We compared the data for which the relevant reference is given.

## Description of the method for two-dimensional TFRDMs

Here, we extend the proposed strategy for two-dimensional TFRDMs^44,45^:

45 $$\begin{aligned} \frac{\partial ^{\alpha } \mathscr {U}(x,y,t)}{\partial t^{\alpha }}&=\varepsilon _{1} \left( \mathscr {U}_{xx}+\mathscr {U}_{yy}\right) - \left( \mathscr {B} +1\right) \mathscr {U} + \mathscr {U}^{2} \mathscr {V} + \mathscr {A} + \mathscr {F}_{1} (x,y,t),\nonumber \\ \frac{\partial ^{\alpha } \mathscr {V}(x,y,t)}{\partial t^{\alpha }}&=\varepsilon _{2} \left( \mathscr {V}_{xx}+\mathscr {V}_{yy}\right) +\mathscr {B} \mathscr {U} - \mathscr {U}^{2} \mathscr {V} +\mathscr {F}_{2} (x,y,t), \end{aligned}$$

where $\varepsilon _{1}, \varepsilon _{2}, \mathscr {A}$ and $\mathscr {B}$ are taken as given in^44,45^, and $\mathscr {F}_{1}$ and $\mathscr {F}_{2}$ are the source functions to be determined via exact solutions. The spatial domain for this problem is $\Omega =[x_0, x_{{\mathbb {N}}}]\times [y_0, y_{{\mathbb {N}}}],$ along with the following ICs and BCs:

46 $$\begin{aligned} \mathscr {U}(x,y,0)= & {} \mathscr {U}_{0}(x,y), \mathscr {V}(x,y,0)=\mathscr {V}_{0}(x,y)\quad x,y \in \Omega . \end{aligned}$$

47 $$\begin{aligned} \mathscr {U}(x_{0},y,t)= & {} \varepsilon _{0}(y,t),\quad \mathscr {U}(x_{{\mathbb {N}}},y,t)=\delta _{0}(y,t), \nonumber \\ \mathscr {U}(x,y_{0},t)= & {} \omega _{0}(x,t),\quad \mathscr {U}(x,y_{N},t) =\xi _{0}(x,t), \quad t > 0 \nonumber \\ \mathscr {V}(x_{0},y,t)= & {} \varepsilon _{1}(y,t),\quad \mathscr {V}(x_{{\mathbb {N}}},y,t)=\delta _{1}(y,t), \nonumber \\ \mathscr {V}(x,y_{0},t)= & {} \omega _{1}(x,t),\quad \mathscr {V}(x,y_{{\mathbb {N}}},t)=\xi _{1}(x,t). \end{aligned}$$

Using the stratagem discussed earlier, Eq. (45) reduces to:

48 $$\begin{aligned}{} & {} \frac{\partial ^{\alpha } \mathscr {U}}{\partial t^{\alpha }}=\varepsilon _{1} \left( \frac{\partial ^{2} \mathscr {U}}{\partial x^{2}}+\frac{\partial ^{2} \mathscr {U}}{\partial y^{2}}\right) ^{n+1} - \left( \beta +1\right) \left( \mathscr {U}\right) ^{n+1} - \left( \mathscr {U}^{2} \mathscr {V}\right) ^{n+1} + \mathscr {A} +\left( \mathscr {F}_{1}\right) ^{n+1}, \end{aligned}$$

49 $$\begin{aligned}{} & {} \frac{\partial ^{\alpha } \mathscr {V}}{\partial t^{\alpha }}=\varepsilon _{2} \left( \frac{\partial ^{2} \mathscr {V}}{\partial x^{2}}+\frac{\partial ^{2} \mathscr {V}}{\partial y^{2}}\right) ^{n+1} + \beta \left( \mathscr {U}\right) ^{n+1} - \left( \mathscr {U}^{2} \mathscr {V}\right) ^{n+1} +\left( \mathscr {F}_{2}\right) ^{n+1}, \end{aligned}$$

where $$ \left( \mathscr {U}\right) ^{n}=\mathscr {U}(x,y,t^n)$$, $$\left( \mathscr {V}\right) ^{n}=\mathscr {V}(x,y,t^n)$$, $$\left( \mathscr {F}_{1}\right) ^{n}=\mathscr {F}_{1}(x,y,t^n)$$ and $$\left( \mathscr {F}_{2}\right) ^{n}=\mathscr {F}_{2}(x,y,t^n).$$

Plugging the quadrature rule for fractional derivative in Eq. (48) and Eq. (49) the new equations are given below:

50 $$\begin{aligned}{} & {} \mathscr {A}_{\alpha } \sum _{k=0}^n \beta ^{\alpha }_k \left[ \left( \mathscr {U}\right) ^{n-k+1} - \left( \mathscr {U}\right) ^{n-k}\right] =\varepsilon _{1} \left( \frac{\partial ^{2} \mathscr {U}}{\partial x^{2}}+\frac{\partial ^{2} \mathscr {U}}{\partial y^{2}}\right) ^{n+1} - \left( \beta +1\right) \left( \mathscr {U}\right) ^{n+1} - \left( \mathscr {U}^{2} \mathscr {V}\right) ^{n+1}\nonumber \\{} & {} \qquad +\mathscr {A} +\left( \mathscr {F}_{1}\right) ^{n+1}, \end{aligned}$$

51 $$\begin{aligned}{} & {} \mathscr {A}_{\alpha } \sum _{k=0}^n \beta ^{\alpha }_k \left[ \left( \mathscr {V}\right) ^{n-k+1} - \left( \mathscr {V}\right) ^{n-k}\right] \nonumber \\{} & {} \quad =\varepsilon _{2} \left( \frac{\partial ^{2} \mathscr {V}}{\partial x^{2}}+\frac{\partial ^{2} \mathscr {V}}{\partial y^{2}}\right) ^{n+1} \nonumber \\{} & {} \qquad + \beta \left( \mathscr {U}\right) ^{n+1} - \left( \mathscr {U}^{2} \mathscr {V}\right) ^{n+1} +\left( \mathscr {F}_{2}\right) ^{n+1}. \end{aligned}$$

After one term expansion Eqs. (6.6–6.7) transform to:

52 $$\begin{aligned}{} & {} \mathscr {A}_{\alpha } \left[ \left( \mathscr {U}\right) ^{n+1} - \left( \mathscr {U}\right) ^{n}\right] + \mathscr {A}_{\alpha } \sum _{k=1}^n \beta ^{\alpha }_k \left[ \left( \mathscr {U}\right) ^{n-k+1} - \left( \mathscr {U}\right) ^{n-k}\right] \nonumber \\{} & {} \quad =\varepsilon _{1} \left( \frac{\partial ^{2} \mathscr {U}}{\partial x^{2}}+\frac{\partial ^{2} \mathscr {U}}{\partial y^{2}}\right) ^{n+1} - \left( \beta +1\right) \left( \mathscr {U}\right) ^{n+1} \nonumber \\{} & {} \qquad -\left( \mathscr {U}^{2} \mathscr {V}\right) ^{n+1} + \mathscr {A} +\left( \mathscr {F}_{1}\right) ^{n+1}, \end{aligned}$$

53 $$\begin{aligned}{} & {} \mathscr {A}_{\alpha } \left[ \left( \mathscr {V}\right) ^{n+1} - \left( \mathscr {V}\right) ^{n}\right] + \mathscr {A}_{\alpha } \sum _{k=1}^n \beta ^{\alpha }_k \left[ \left( \mathscr {V}\right) ^{n-k+1} - \left( \mathscr {V}\right) ^{n-k}\right] \nonumber \\{} & {} \quad =\varepsilon _{2} \left( \frac{\partial ^{2} \mathscr {V}}{\partial x^{2}}+\frac{\partial ^{2} \mathscr {V}}{\partial y^{2}}\right) ^{n+1} + \beta \left( \mathscr {U}\right) ^{n+1} \nonumber \\{} & {} \qquad -\left( \mathscr {U}^{2} \mathscr {V}\right) ^{n+1} +\left( \mathscr {F}_{2}\right) ^{n+1}. \end{aligned}$$

Rearranging the terms in the above system the results are:

54 $$\begin{aligned}{} & {} \varepsilon _{1} \left( \frac{\partial ^{2} \mathscr {U}}{\partial x^{2}}+\frac{\partial ^{2} \mathscr {U}}{\partial y^{2}}\right) ^{n+1} - \left( \beta +1\right) \left( \mathscr {U}\right) ^{n+1} - \left( \mathscr {U}^{2} \mathscr {V}\right) ^{n+1} -\mathscr {A}_{\alpha } \left( \mathscr {U}\right) ^{n+1}= -\left( \mathscr {U}\right) ^n -\left( \mathscr {F}_{1}\right) ^{n+1} \nonumber \\{} & {} \quad -\mathscr {A}+ \mathscr {A}_{\alpha } \sum _{k=1}^n \beta ^{\alpha }_k \left[ \left( \mathscr {U}\right) ^{n-k+1} - \left( \mathscr {U}\right) ^{n-k}\right] , \end{aligned}$$

55 $$\begin{aligned}{} & {} \varepsilon _{2} \left( \frac{\partial ^{2} \mathscr {V}}{\partial x^{2}}+\frac{\partial ^{2} \mathscr {V}}{\partial y^{2}}\right) ^{n+1} + \beta \left( \mathscr {U}\right) ^{n+1} - \left( \mathscr {U}^{2} \mathscr {V}\right) ^{n+1} -\mathscr {A}_{\alpha } \left( \mathscr {V}\right) ^{n+1}=-\left( \mathscr {V}\right) ^n -\left( \mathscr {F}_{2}\right) ^{n+1} \nonumber \\{} & {} \quad +\mathscr {A}_{\alpha } \sum _{k=1}^n \beta ^{\alpha }_k \left[ \left( \mathscr {V}\right) ^{n-k+1} -\left( \mathscr {V}\right) ^{n-k}\right] . \end{aligned}$$

Linearization of the nonlinear term $\left( \mathscr {U}^{2} \mathscr {V}\right) ^{n+1}$ is tackled by the following formula^42^:

56 $$\begin{aligned} \left( \mathscr {U}^{2} \mathscr {V}\right) ^{n+1} = 2 \left( \mathscr {U}\right) ^{n} \left( \mathscr {V}\right) ^{n} \left( \mathscr {U}\right) ^{n+1} -2 \left( \mathscr {U}^{2}\right) ^{n} \left( \mathscr {V}\right) ^{n} +\left( \mathscr {U}^{2}\right) ^{n} \left( \mathscr {V}\right) ^{n+1} \end{aligned}$$

Inserting the approximation of the nonlinear term, and the central difference approximation for the involved derivative, the linear system of equations can be obtained which are given in compact form:

$$\begin{aligned} \mathscr {A}\mathscr {W}^{n+1}=\mathscr {W}^{n}, \end{aligned}$$

where $\mathscr {A}$=${(2\mathscr {M}-2)^2} \times {(2\mathscr {M}-2)^2}$ and $\mathscr {W}^{n+1} = (2 \mathscr {M}-2)^2\times 1$ matrices.

**Figure 11:**
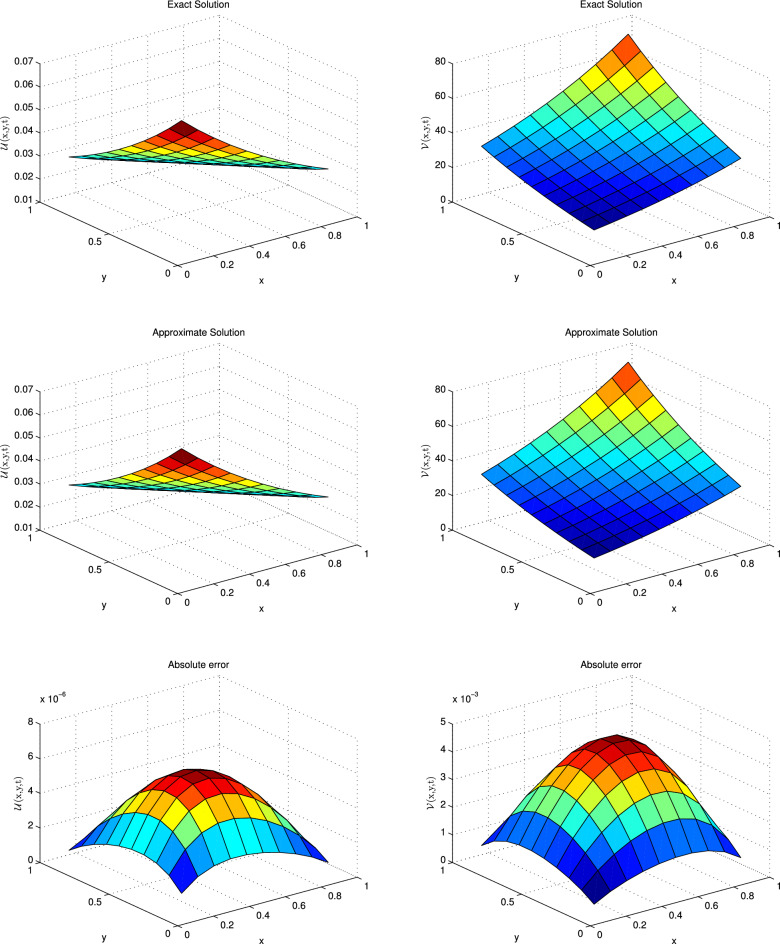
Plotting of $\mathscr {U}$ and $\mathscr {V}$ at t = 5 when $\alpha =1$ and $dt=0.001$.

**Figure 12:**
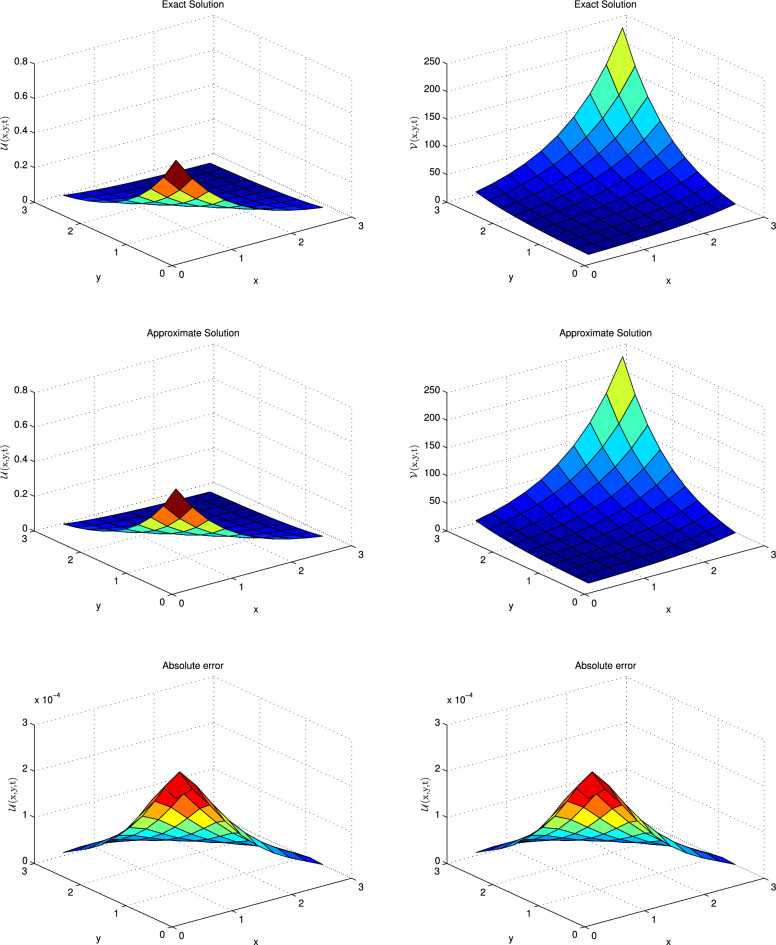
Solution profiles of $\mathscr {U}$ and $\mathscr {V}$ at t=0.1 when $\alpha =0.5$ and $dt=0.001$.

### Problem 5.4 (2D Brusselator model)

Here, the following two-dimensional Brusselator model of kinetics for two chemical components is considered for validation of the scheme^44,45^:

57 $$\begin{aligned} \frac{\partial ^{\alpha } \mathscr {U}(x,y,t)}{\partial t^{\alpha }}&=\varepsilon _{1} \left( \mathscr {U}_{xx}+\mathscr {U}_{yy}\right) -\left( \mathscr {B} +1\right) \mathscr {U} + \mathscr {U}^{2} \mathscr {V} + \mathscr {A} + \mathscr {F}_{1} (x,y,t),\nonumber \\ \frac{\partial ^{\alpha } \mathscr {V}(x,y,t)}{\partial t^{\alpha }}&=\varepsilon _{2} \left( \mathscr {V}_{xx}+\mathscr {V}_{yy}\right) + \mathscr {B} \mathscr {U} - \mathscr {U}^{2} \mathscr {V} +\mathscr {F}_{2} (x,y,t), \end{aligned}$$

along with the following conditions:

58 $$\begin{aligned}{} & {} \mathscr {U}(x,y,0)=e^{ (-x-y)} \ \, \ \ \mathscr {V}(x,y,0)=e^{ (x+y)},\quad x,y \in \Omega . \end{aligned}$$

59 $$\begin{aligned}{} & {} \mathscr {U}(0,y,t) =e^{ (-y-0.5t)},\qquad \mathscr {U}(1,y,t)=e^{ (-1-y-0.5t)}, \nonumber \\{} & {} \mathscr {U}(x,0,t) =e^{ (-x-0.5t)}),\qquad \mathscr {U}(x,1,t) =e^{ (-x-1-0.5t)}, \quad t > 0 \nonumber \\{} & {} \mathscr {V}(0,y,t) =e^{ (y + 0.5t)}, \qquad \mathscr {V}(1,y,t)=e^{ (1+y+0.5t)}, \nonumber \\{} & {} \mathscr {V}(x,0,t) =e^{ (x + 0.5t)},\qquad \mathscr {V}(x,1,t)=e^{ (x+1+0.5t)}. \quad \end{aligned}$$

**Table 12:** $\mathbb {L}_{\infty }$ norms for U and V at t = 2 and dt = 0.001 of Brusselator model in 2D for $\alpha =1.$.

dx	proposed method $\mathbb {L}_{\infty }$		proposed method $\mathbb {L}_{2}$		proposed method *RMS*		^45^ $\mathbb {L}_{\infty }$		^46^ $\mathbb {L}_{\infty }$	
	$\mathscr {U}$	$\mathscr {V}$	$\mathscr {U}$	$\mathscr {V}$	$\mathscr {U}$	$\mathscr {V}$	$\mathscr {U}$	$\mathscr {V}$	$\mathscr {U}$	$\mathscr {V}$
1/10	2.7029e−05	9.6987e−04	1.6094e−04	5.9584e−03	1.6094e−05	5.9584e−04	2.7094e−05	1.7571e−03	7.6449e−05	3.6792e−03
1/20	1.3277e−05	4.7984e−04	1.4357e−04	5.3227e−03	7.5564e−06	2.8014e−04	2.3714e−05	7.5561e−04	2.3469e−05	1.2749e−03
1/25	1.0147e−05	3.4828e−04	1.3718e−04	4.8381e−03	5.7160e−06	2.0159e−04	1.3115e−05	1.4635e−03	1.5346e−05	8.3510e−04

The associated source terms can be adjusted using the exact solution and the formula:

$$\begin{aligned} ~^{\mathscr {C}}_{0}\mathscr {D}_{x}^{\alpha }(e^{\lambda x}) =\lambda ^{n} x^{n-\alpha } \mathbb {E}_{1,n-\alpha +1}(\lambda x), \end{aligned}$$

where $\mathbb {E}_{1,n-\alpha +1}(:)$ is the Mittag-Leffler function defined earlier. The closed-form solution to the above problem is:

$$\begin{aligned} \mathscr {U}(x,y,t)= & {} e^{ (-x-y-0.5t)},\\ \mathscr {V}(x,y,t)= & {} e^{(x+y+0.5t)}. \end{aligned}$$

We solve this model for the parameters $\varepsilon _{1}= \varepsilon _{2}=0.25, \mathscr {A}=0, \mathscr {B}=1, dx=dt$. Also, the spatial and temporal domains are $[0, 1]\times [0, 1],$ and [0,5], respectively. In Table 12 the obtained error norms are matched with the previous norms given in the papers for integer case^44,45^. It is noticed that computed outcomes are matchable with available solutions. Further, the scheme is tested for fractional values of $\alpha$ and the achieved results are reported in the form of ${\mathbb {L}}_{\infty }$ and ${\mathbb {L}}_{2}$ norms in Tables 13 and 14, respectively. From these tables one can see, that the proposed scheme works for fractional cases as well. For further clarification, the solutions are sketched for integer and fractional cases in Figs. 11 and 12, which show that exact and numerical solutions are promised well.

**Table 13:** $\mathbb {L}_\infty$ norm of Brusselator model in 2D for different $\alpha$ and time.

t	$\alpha =0.25$		$\alpha =0.5$		$\alpha =0.75$		$\alpha =1$	
	$\mathbb {L}_{\infty }$ of $\mathscr {U}$	$\mathbb {L}_{\infty }$ of $\mathscr {V}$	$\mathbb {L}_{\infty }$ of $\mathscr {U}$	$\mathbb {L}_{\infty }$ of $\mathscr {V}$	$\mathbb {L}_{\infty }$ of $\mathscr {U}$	$\mathbb {L}_{\infty }$ of $\mathscr {V}$	$\mathbb {L}_{\infty }$ of $\mathscr {U}$	$\mathbb {L}_{\infty }$ of $\mathscr {V}$s
0.001	9.6134e−05	3.6464e−02	1.6767e−05	6.6861e−03	6.5949e−06	2.6495e−03	1.7943e−06	7.2387e−04
0.01	1.4283e−04	5.2265e−02	4.7277e−05	1.8750e−02	3.4592e−05	1.3754e−02	1.7389e−05	7.0160e−03
0.05	2.1876e−04	7.7603e−02	1.2651e−04	4.9876e−02	1.3946e−04	5.5138e−02	7.6450e−05	3.0925e−02
0.075	2.5953e−04	9.1730e−02	1.9894e−04	7.4616e−02	2.2068e−04	8.7199e−02	1.0665e−04	4.3279e−02
0.1	3.0504e−04	1.0727e−01	2.9193e−04	1.0733e−01	3.2034e−04	1.2444e−01	1.3295e−04	5.4176e−02

**Table 14:** $\mathbb {L}_{2}$ norm of Brusselator model in 2D for different $\alpha$ and time.

t	$\alpha =0.25$		$\alpha =0.5$		$\alpha =0.75$		$\alpha =1$	
	$\mathbb {L}_{2}$ of $\mathscr {U}$	$\mathbb {L}_{2}$ of $\mathscr {V}$	$\mathbb {L}_{2}$ of $\mathscr {U}$	$\mathbb {L}_{2}$ of $\mathscr {V}$	$\mathbb {L}_{2}$ of $\mathscr {U}$	$\mathbb {L}_{2}$ of $\mathscr {V}$	$\mathbb {L}_{2}$ of $\mathscr {U}$	$\mathbb {L}_{2}$ of $\mathscr {V}$
0.001	3.0527e−04	1.1432e−01	4.2678e−05	1.6998e−02	1.6061e−05	6.4519e−03	4.2605e−06	1.7188e−03
0.01	5.1760e−04	1.8633e−01	1.3539e−04	5.3386e−02	9.3857e−05	3.7239e−02	4.2242e−05	1.7042e−02
0.05	8.1083e−04	2.8673e−01	4.5437e−04	1.7421e−01	4.5566e−04	1.7771e−01	2.0426e−04	8.2508e−02
0.075	9.7574e−04	3.4300e−01	7.3575e−04	2.7506e−01	7.6843e−04	2.9778e−01	3.0099e−04	1.2176e−01
0.1	1.1632e−03	4.0580e−01	1.0933e−03	3.9948e−01	1.1542e−03	4.4491e−01	3.9502e−04	1.6010e−01

## Conclusions and future plan

In this work, an implicit scheme has been addressed to solve RDCMs in fractional form. The involved fraction derivative and spatial derivatives were approximated with a well-known $L_{1}$ formula (Quadrature rule) and finite differences, respectively. Next, the stability of the scheme was investigated via Von Neumann analysis. Moreover, the scheme has been tested with different linear and nonlinear problems and the outcomes were compared with exact and existing results in literature. From tabulated simulations and graphical solutions, it has been observed that the proposed scheme works well for RDMs and can be used for such complicated problems having no exact solutions. In future the proposed methodology can be extended to the three dimensional problems coupling with different variable order fractional derivatives like, Caputo Fabrizio, and Atangana–Baleanu–Caputo etc. Moreover, the strategy can be tested for variable order local fractional derivative problems as well.

## Data Availability

All data generated or analysed during this study are included in this published article.
